# A biodegradable capacitive-coupling neurostimulator for wireless electroceutical treatment of inflammatory bowel diseases

**DOI:** 10.1126/sciadv.adu5887

**Published:** 2025-02-14

**Authors:** Qiong Wang, Ming Yang, Renyuan Sun, Wenliang Liu, Wenlong Li, Baochun Xu, Shiming Yang, Ke Chen, Jun Xiao, Xuyong Chen, Xinyao Meng, Jiexiong Feng, Cunjiang Yu, Zhiqiang Luo

**Affiliations:** ^1^ Department of Pediatric Surgery, Tongji Hospital, Tongji Medical College, Huazhong University of Science and Technology, Wuhan, Hubei 430030, China.; ^2^ National Engineering Research Center for Nanomedicine, College of Life Science and Technology, Huazhong University of Science and Technology, Wuhan, Hubei 430074, China.; ^3^ Materials Research Laboratory, University of Illinois, Urbana-Champaign, Urbana, IL 61801, USA.; ^4^ Hubei Clinical Center of Hirschsprung's Disease and Allied Disorders, Wuhan, Hubei 430030, China.; ^5^ Department of Electrical and Computer Engineering, Department of Materials Science and Engineering, Department of Mechanical Science and Engineering, and Department of Bioengineering, Beckman Institute for Advanced Science and Technology, Nick Holonyak Micro and Nanotechnology Laboratory, University of Illinois, Urbana-Champaign, Urbana, IL 61801, USA.

## Abstract

Electroceuticals based on peripheral nerve stimulation offer promising treatment for refractory inflammatory diseases such as inflammatory bowel diseases (IBDs). For pediatric IBD (PIBD) patients, wireless, biodegradable miniaturized bioelectronic devices are crucial to prevent neural damage and support neural development during and after therapy. Here we demonstrate a battery-free, miniaturized neurostimulator based on biodegradable materials and capacitive-coupling wireless power transfer. The biodegradable capacitive-coupling (BCC) neurostimulator consists of molybdenum (Mo) electronic components and self-healing biodegradable polyurethane elastomer (SBPUE) encapsulations. The self-healing property of SBPUE enables a stable neural interface. Capacitive coupling wirelessly transfers high-frequency electric fields through a single capacitor between wearable transmitters and implanted BCC neurostimulators. Programmed electrical stimulation of the vagus nerve alleviates PIBD symptoms by restoring CD4^+^ T cell balance, enhancing anti-inflammatory effects and suppressing pro-inflammatory effects in the intestines.

## Teaser

Biodegradable capacitive-coupling neurostimulator enables wireless therapy, restoring immune balance in pediatric IBD.

## INTRODUCTION

Pediatric inflammatory bowel disease (PIBD), an immune-mediated disorder of the gastrointestinal tract, is a refractory disease without effective treatment (*1*). The incidence of PIBD has been increasing, and children affected by this condition often face additional complications such as growth impairment, delayed puberty, psychological issues, and concerns regarding body image (*2*, *3*). Current drug therapies and surgical interventions offer limited success; therefore, there is an urgent search for more effective treatments. Recently, electroceuticals based on bioelectronic devices for peripheral stimulation have been explored in preclinical research to treat refractory metabolic and immune-mediated diseases (*4*, *5*). Electrical stimulation, particularly vagus nerve stimulation (VNS), has been shown to modulate the autonomic nervous system, thereby reducing the levels of pro-inflammatory cytokines such as tumor necrosis factor–α (TNF-α). This intervention enhances gastrointestinal motility and function while alleviating extraintestinal symptoms via brain-gut axis signaling (*6*–*9*). Beyond physical symptom relief, electrical stimulation offers the potential to reduce reliance on medications, minimizing associated side effects, and to address the psychological burdens of PIBD, including anxiety and depression, which are prevalent among children managing chronic illnesses (*10*–*14*). However, existing bioelectronic devices commonly involve nonbiodegradable neural electrodes and bulky power supply systems. These implanted nonbiodegradable components can lead to severe lacerations and perforations of neural tissue upon removal, as they may become enveloped by fibrotic tissues at the electrode-tissue interface. On the other hand, leaving them in place would restrict neural development in children (*15*, *16*). Therefore, there is an urgent need in biodegradable electroceutical devices, particularly for growing pediatric patients whose nerves are still in the developmental stage with a potential size increase.

The limitations of bulky battery powered implants underscore the need for miniaturized, wireless-powered bioelectronic devices (*17*–*20*). Recently, implantable soft piezoelectric and triboelectric nanogenerators, which harvest energy from internal organ movements such as breathing, heartbeats, or muscle stretching, have been investigated for use in self-powered bioelectronics (*21*–*23*). To achieve precise control of stimulation parameters, including current density, pulse width, stimulation frequency, and treatment duration, ultrasound stimulation externally is usually used (*6*, *24*, *25*). Nonetheless, biodegradable ultrasound-driven nanogenerators with a high current output for electroceutical treatment remain a challenge. In addition, the stimulation procedures with bulky ultrasound generators usually necessitate it to take place in a clinical setting. These highlight an ideal solution of a high-performance electroceutical device that combines biodegradable neural electrodes, wiring, and wireless power transfer components in an all-in-one format.

The construction of a robust neural interface is crucial for reliable chronic neurostimulation during long-term electroceutical therapy, and it is challenging for commonly used mouse and rat models with tiny peripheral nerves (*26*, *27*). Commonly used cuff electrodes that wrap around peripheral nerves are fixed through mechanical suturing (*28*, *29*), often leading to stress compression and increased inflammation in nerve tissues. To mitigate the high-stress compression of cuff electrodes on peripheral nerves, researchers have recently investigated advanced fixation methods (*30*–*35*). For example, well-designed mechanical structures, shape memory polymers, and mechanically adaptive hydrogels have been developed for elaborate fastening of cuff electrodes (*36*). Despite these innovative approaches, designing multifunctional materials that provide stress-free fixation of neural electrodes and biodegradable features for transient bioelectronics remains elusive. We envision that the use of biodegradable elastomers with self-healing properties as an encapsulation layer for neural electrodes to eliminate potential suturing could effectively address this challenge, as their self-healing capability enables gentle yet effective fixation.

**Fig. 1.**
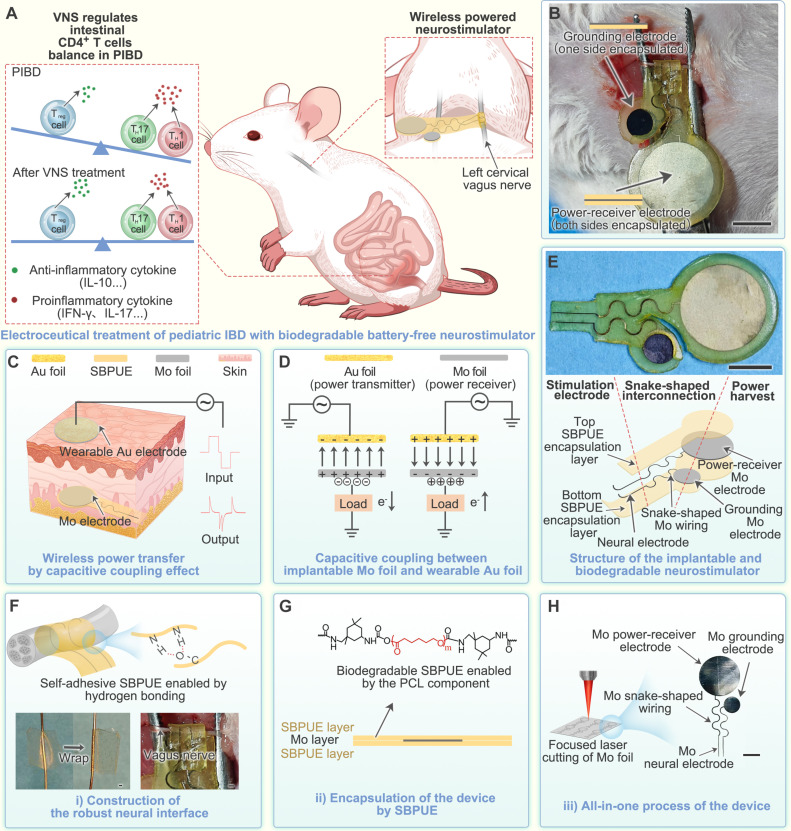
BCC neurostimulator for electroceutical treatment of PIBD. (**A**) Schematic of the battery-free, wirelessly powered BCC neurostimulator for chronic vagus nerve stimulation to regulate intestinal CD4^+^ T cell balance in PIBD. (**B**) Surgical image showing the implantation of the BCC neurostimulator on the rat vagus nerve. Scale bar, 5 mm. (**C**) Wireless power transfer by capacitive coupling. (**D**) Capacitive coupling mechanism. (**E**) Three-layer structure of the BCC neurostimulator. Scale bar, 5 mm. (**F**) Self-healing property of SBPUE enables construction of a robust neural interface. Scale bars, 400 μm (left) and 200 μm (right). (**G**) Self-healing SBPUE enables rapid, seamless device encapsulation at room temperature. (**H**) All-in-one integration of biodegradable Mo neural stimulation electrodes, device wiring, capacitive coupling electrode, and grounding electrode on a single Mo foil using laser cutting technology. Scale bar, 5 mm.

To address these challenges, here we report the development of a biodegradable capacitive-coupling (BCC) neurostimulator capable of electroceutical treatment of PIBD based on capacitive-coupling wireless-power-transfer technology (Fig. 1, A and B). Capacitive coupling, i.e., wireless power transfer through a high-frequency electric field, can be realized with the single capacitor between the wearable power-transmitter electrodes and the implanted BCC neurostimulator (Fig. 1, C and D). The BCC neurostimulator is fully composed of biodegradable and biocompatible components, including all-in-one integrated molybdenum (Mo) electronic components and self-healing biodegradable polyurethane elastomer (SBPUE) encapsulation. The self-healing property of the SBPUE facilitates the easy fixation of neural-stimulation electrodes onto tiny nerves. We validated the capabilities of the BCC neurostimulator in an in vivo rat PIBD model. Chronic stimulation of the vagus nerve can effectively modulate the immune microenvironment by restoring the balance of intestinal CD4^+^ T cells, which promotes anti-inflammatory effects and inhibits pro-inflammatory effects. Our BCC neurostimulator was also shown to reduce the outcomes of PIBD complications, including weight loss, colon shortening, increased intestinal permeability, and enlargement of the mesenteric lymph nodes, thereby enhancing overall intestinal health. The associated materials and device designs, biomedical efficacy validations, and mechanistic understandings collectively highlight the innovation and significances of such BCC neurostimulator.

## RESULTS

### Construction and device performance of the BCC neurostimulator

The BCC neurostimulator was constructed using a three-layer design (Fig. 1E). The outermost layer was composed of a ~100-μm-thick SBPUE layer, which acted as a flexible and self-healing substrate for constructing a robust neural interface (Fig. 1F). In addition, the SBPUE layer served as a biodegradable encapsulation, enhancing the biocompatibility and safety of the device in biological environments (Fig. 1G). The middle layer was a laser-patterned Mo foil (~20 μm thick) that enabled the all-in-one integration of neural-stimulation electrodes, device-wiring, power-receiver electrode, and grounding electrode (Fig. 1H). The implanted Mo power-receiver electrode, with a diameter of 1 cm, was encapsulated on both sides to provide insulation and protection. Meanwhile, the implanted grounded Mo electrode was encapsulated on one side only, allowing direct contact with the rat’s neck skin tissue. For capacitive-coupling wireless power delivery to the BCC stimulator, a wearable gold (Au) power-transmitter electrode was aligned with the capacitive-coupling Mo power-receiver electrode. The wearable grounding electrode (encapsulated on one side) and implanted grounding Mo electrode were electrically connected through the skin tissue to ensure stable grounding. For better understanding of the capacitive-coupling wireless power delivery system, the detailed labels for its main components are illustrated in fig. S1.

**Fig. 2.**
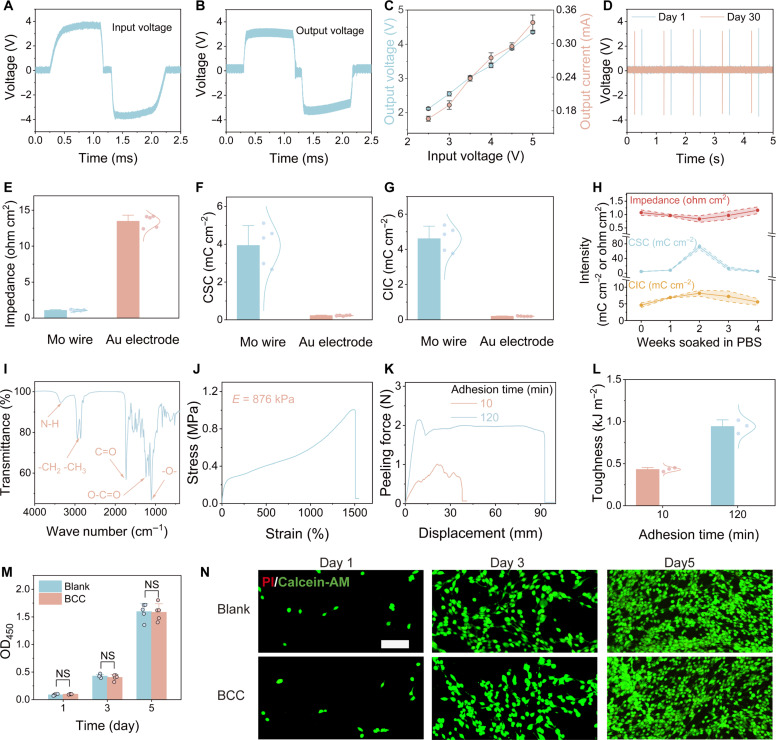
Device performances and biosafety evaluation of the BCC neurostimulator. (**A**) Input voltage waveform of the power-transmitter Au-foil electrode. (**B**) Output voltage pulse generated by the power-receiver Mo-foil electrode. (**C**) Open-circuit voltage and short-circuit current generated by the BCC neurostimulator. (**D**) Output voltage stability of the BCC neurostimulator during 4-week immersing in PBS at 37°C. (**E**) Impedance comparison of Mo and Au electrodes at 1 kHz (*n* = 5). (**F**) CSC property of Mo and Au electrodes (*n* = 5). (**G**) CIC property of Mo and Au electrodes (*n* = 5). (**H**) Long-term electrochemical monitoring of Mo electrodes in PBS at 37°C (*n* = 5). (**I**) FT-IR spectrum of the SBPUE. (**J**) Representative tensile stress-strain curve of the SBPUE. (**K**) Representative load-displacement curves of two self-healed SBPUE. (**L**) Interfacial toughness of self-healed SBPUE (*n* = 3). (**M**) Viability of PC12 cells cocultured with the SBPUE extracts at 1, 3, and 5 days (*n* = 5). (**N**) Representative live-dead staining images of PC12 cells with the SBPUE extracts at 1, 3, and 5 days. Data are presented as the mean ± SD in (C), (E), (F), (G), (H), (L), and (M). Scale bar, 100 μm.

Biphasic stimulation is vital in biomedical stimulators because it uses charge-balanced waveforms to prevent charge accumulation and averts deleterious electrochemical reactions at the electrode interface. Traditional methods of wireless energy transfer, such as optoelectronic effects and inductive coupling, generally require elaborate circuitry to realize biphasic stimulation (*37*, *38*). By leveraging the capacitive coupling mechanism based on electrostatic induction, adjustable biphasic electrical pulses with a precisely controlled input voltage waveform can be generated. The input voltage waveform was generated by a trapezoidal wave with a sinusoidal carrier waveform, and the edited waveform was used to produce biphasic rectangular pulses (±4 V, 1 Hz, 0.6 ms per phase) with an interval of 0.15 ms (between two phases) (Fig. 2A). In response to the voltage input of the wearable power-transmitter Au-foil electrodes, the power-receiver Mo-foil electrode in the BCC neurostimulator efficiently output biphasic charge-balanced rectangular voltage pulses with an amplitude of approximately ±3.38 V (Fig. 2B), and the corresponding output short-circuit current was approximately 250 μA (fig. S2). As shown in Fig. 2C, the short-circuit current and open-circuit voltage increased with the input voltage. Therefore, the desired output for neuromodulation can be obtained by adjusting the input voltage applied to the Au power-transmitter foils. We also evaluated the impact of electrode shape and size (fig. S3, A and B) and determined that the selected dimensions are optimal based on experimental results and in vivo size requirements. To assess the influence of tissue thickness, the tissue resistance (fig. S3C) and the overall system impedance (fig. S3D) were used to calculate the voltage transfer efficiency (fig. S3E). Experimental validation further demonstrated that variations in distance between the power-transmitter Au foil and power-receiver Mo foil had an in notable effect on capacitive-coupling efficiency (fig. S3F). In addition, the system showed tolerance to minor electrode misalignments (fig. S3G). As for the long-term stability of capacitive coupling efficiency, open-circuit voltage measurements were conducted during 30 days of immersion in phosphate-buffered saline (PBS) at 37°C, and excellent coupling stability was observed (Fig. 2D and fig. S3H).

To achieve the maximum output at the power-receiver end with an in vivo load (neural-stimulation electrodes connected to vagus nerve tissues), we further studied the impedance matching by measuring and fitting the resistance and reactance in the nerve tissues, as well as the capacitance of capacitive coupling at varying frequencies (fig. S4). On the basis of the calculation, a 1-mH inductor was incorporated into the wearable power-transmitter circuit to achieve reactance matching under high-frequency conditions. Because the resonant frequency of the capacitive coupling with load mimicking nerve tissues is about 1 MHz, a 1-MHz sine wave with an amplitude of 0.4 V was used as the carrier during waveform editing. We then investigated the relationship between the current output of Mo power-receiver electrodes and the loading resistances under different input voltages (fig. S5). The output current of the Mo power-receiver electrode decreased as the loading resistance increased from 2000 to 20,000 ohms. With reactance matching, the current output reached approximately 250 μA when the load resistance was 10,000 ohms (corresponding to the impedance of vagus nerve tissues at 1 MHz). The output voltage traces with and without reactance matching are shown in fig. S6. With reactance matching, the output voltage of the implanted power-receiver device can be substantially improved. These results underscore the importance of reactance matching between the power-transmission and the power-receiver end in ensuring more efficient capacitive coupling.

The electrochemical properties of biodegradable Mo electrodes were thoroughly investigated to assess their neuromodulation capabilities. Au electrodes, which are commonly used in bioelectronics, were also examined for comparison. The Mo electrodes exhibited a lower charge transfer resistance than the Au electrodes (Fig. 2E), lowering the impedance at the interface between the electrode and nerve tissue (fig. S7A). The charge storage and exchange capacities were further examined to determine their sensitivity to external electrical signals. The Mo electrodes showed a larger charge storage capability (CSC) (3.93 ± 1.06 mC·cm^−2^) than that of Au electrodes (0.22 ± 0.027 mC·cm^−2^; Fig. 2F and fig. S7B). Under electrical stimulation with biphasic pulses (±0.5 V, 1.5 ms), the Mo electrodes realized a much higher current density of ~500 mA cm^−2^ with a stimulation voltage of ±0.5 V (fig. S7C). The Mo electrodes had a charge injection capability (CIC) of 4.6 ± 0.71 mC cm^−2^, which was considerably higher than that of Au electrodes (0.199 ± 0.017 mC cm^−2^) (Fig. 2G). The long-term stability of impedance, CSC, and CIC of the Mo electrodes was also investigated. The impedance of the Mo electrodes showed a decreasing trend during the first 2 weeks and an increasing trend in the following 2 weeks, while CSC and CIC values were opposite, first increasing and then decreasing (Fig. 2H). Mo neural-stimulation electrodes with enhanced electrochemical properties show the potential to support low-intensity and high-efficiency electrical stimulation for safe neuromodulation.

### Encapsulation of the BCC neurostimulator

The encapsulation layer of the device is a critical component of the neurostimulator. Thus, the rational synergistic molecular design of SBPUE was explored. Specifically, SBPUE used polycaprolactone (PCL) and poly(tetrahydrofuran) (PTHF) as soft segments and dimethylglyoxime as hard segments (fig. S8). The soft segments of SBPUE contributed to its low crystallinity, excellent stretchability, degradability, and good cytocompatibility, whereas the hard segments increased their toughness and self-healing capability. The Fourier transform infrared spectrum of SBPUE was analyzed (Fig. 2I). The peak at 1105 cm^−1^ indicates ether functional groups, whereas the peaks at 1238 and 1732 cm^−1^ represent ester functional groups. In addition, the peaks at 2864 and 3372 cm^−1^ correspond to -CH_2_-CH_3_ and N-H stretching vibrations, respectively. These findings were corroborated by the nuclear magnetic resonance analysis (fig. S8).

We further investigated the mechanical and self-healing properties of SBPUE. The SBPUE demonstrated extreme elasticity, accommodating linear strains of up to approximately 1500% (fig. S9A), with a tensile modulus measured at 876 kPa (Fig. 2J). These results indicate weaker intermolecular forces and enhanced stretchability of the molecular chains, attributed to the mixed soft segments of PCL and PTHF. The hysteresis of the elastic recovery of the SBPUE was evaluated by applying 10 cycles of 20% strain. The SBPUE did not exhibit perfect recovery in the first loading-unloading cycle, with a residual strain of approximately 15% after the tensile stress returned to zero (fig. S9B). The tensile stress loss in the subsequent cycles was slightly lower than that in the earlier cycles, which can be partially attributed to the energy dissipation resulting from the dissociation of hydrogen bonds. We also measured the compressive modulus and shear modulus of SBPUE, which were 81 kPa (fig. S9C) and 1.22 MPa (fig. S9D), respectively. The substantially lower compressive modulus may be related to the relatively loose molecular chains of the elastomer, while the higher shear modulus could be attributed to the easier sliding or relative movement between the molecular chains. The stress-strain curve of the serpentine molybdenum wire (fig. S9, E and F) revealed a fracture strain of 55%, demonstrating the device’s excellent extensibility for in vivo applications. In addition, SBPUE demonstrated fast self-healing owing to the multiple hydrogen bonds within it. The scratches on the SBPUE film self-healed at room temperature within 120 min (fig. S10). The peeling test of two self-healed SBPUE films demonstrated strong adhesion (Fig. 2K and fig. S11), measuring 420.8 ± 22.63 J m^−2^ after a 10-min healing period, and increased to 937 ± 111.7 J m^−2^ after a 120-min healing period (Fig. 2L). The lap shear test of two SBPUE films also confirmed their strong self-adhesion capability (fig. S11, B and C). After 10 and 120 min of healing, the adhesion strengths were 37.56 ± 11.19 kPa and 69.08 ± 9.89 kPa, respectively (fig. S11D). These results indicate that SBPUE achieves rapid and complete healing within 120 min, potentially enabling self-healing–based fixation of neural-stimulation electrodes. This approach can replace traditional mechanical suturing methods, ensuring stability and reducing possible tissue damage during fixation.

To verify the biocompatibility of the BCC neurostimulator, we conducted both cell counting kit-8 (CCK-8) and live-dead staining assays to assess its in vitro cytotoxicity. We cultured two nerve cell lines (PC12 and ND7/23) and a mouse fibroblast cell line (L929) with BCC extracts for 5 days, using normal Dulbecco’s modified Eagle’s medium as the control. The relative viabilities of the cells cultured with the BCC extracts were comparable to those of the control group (Fig. 2M and fig. S12, A and B). In addition, there was no noteworthy difference in cell morphology between the BCC extract group and control groups (Fig. 2N and fig. S12, C and D). These results suggest that the BCC neurostimulator is noncytotoxic.

### Functionality of the BCC neurostimulator

**Fig. 3.**
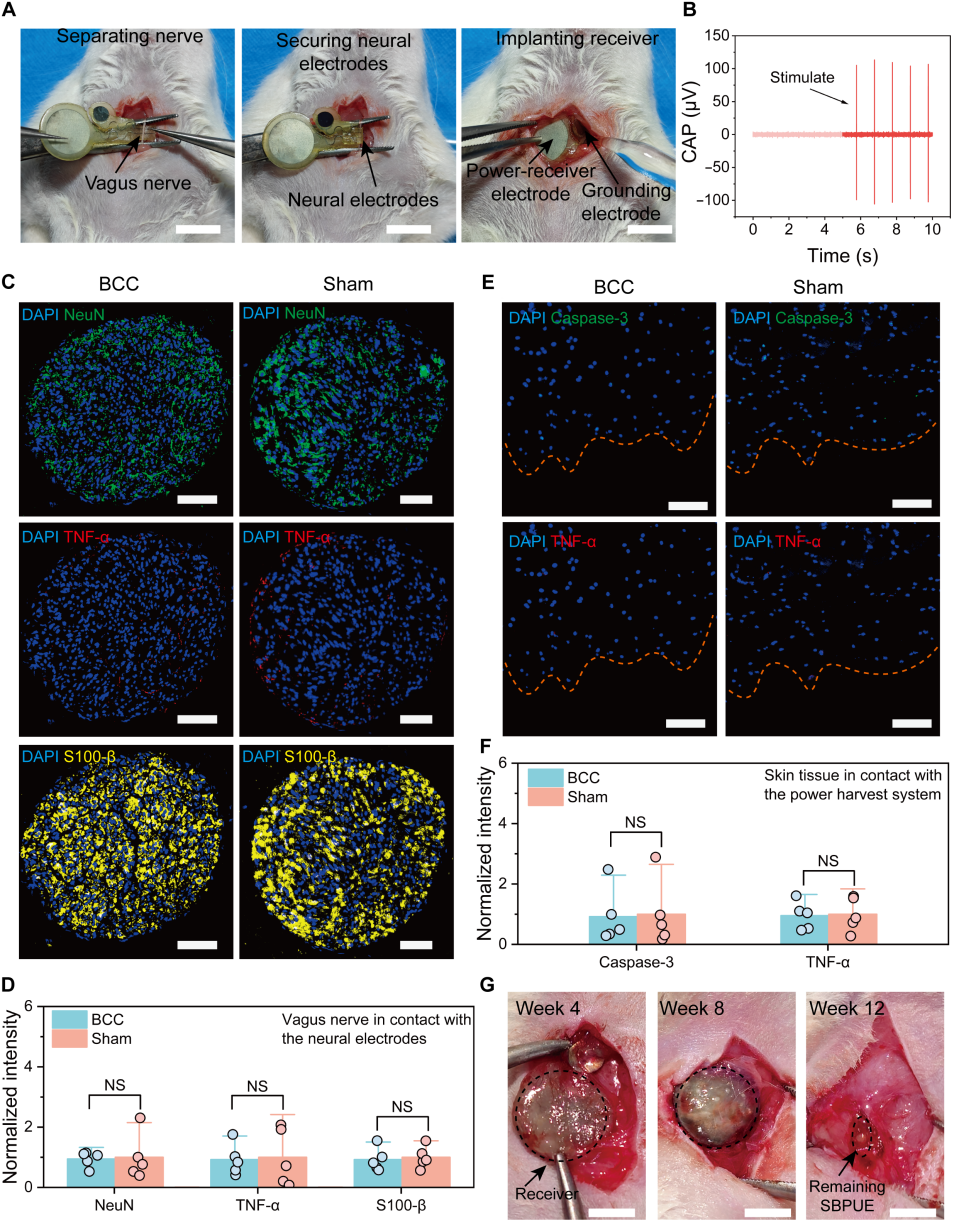
In vivo biocompatibility and biodegradation of the BCC neurostimulator. (**A**) Process of the BCC neurostimulator implantation. Scale bars, 10 mm. (**B**) CAP of the vagus nerve by the BCC neurostimulator. (**C**) Representative immunofluorescence staining of the vagus nerves after 4-week implantation. Scale bars, 50 μm. (**D**) Normalized fluorescence intensity of NeuN, TNF-α, and S-100β from different groups (*n* = 5). (**E**) Representative immunofluorescence staining of the skin tissues after 4-week implantation. Scale bars, 100 μm. (**F**) Normalized fluorescence intensity of caspase-3 and TNF-α from different groups (*n* = 5). (**G**) Biodegradation of implanted BCC neurostimulator within 12 weeks. Scale bars, 5 mm. Data are presented as the mean ± SD in (D) and (F) and were analyzed by one-way ANOVA first, and then by the Tukey′s post hoc test. NS, not significant.

To characterize the functionality of the BCC neurostimulator in a living organism, we tested the BCC neurostimulator with both the vagus and sciatic nerves in the rat model. The capacitive-coupling powering electrodes consisted of a pair of soft insulated Mo foils placed under the neck skin, serving as the power-receiver electrode (encapsulated on both sides) and grounding electrode (encapsulated on one side) (Fig. 3A). On top of the neck skin, we placed an Au foil, which served as the power-transmitter electrode (encapsulated on both sides), and placed a small external grounding electrode (Mo foil encapsulated on a single side) (fig. S13, A and B). The external grounding electrode and implanted grounding electrode were both electrically connected to the skin tissue, and the external power-transmitter electrode was aligned with the implanted power-receiver electrode (fig. S13C). The capacitive-coupling electrical stimulation pulses (power-transmitter input of ±4 V, pulse width of 0.6 ms per phase, and stimulation frequency of 1 Hz) could induce compound action potential (CAP) of the vagus nerves without notable temperature elevation during 20-min capacitive-coupling electrical stimulation (Fig. 3B and fig. S14). Similarly, capacitive-coupling electrical-stimulation pulses could induce sciatic nerve response (fig. S15A), characterized by compound muscle action potential (CMAP) and gastrocnemius tension changes (fig. S15, B and C, and movie S1).

We also investigated the immune responses in nerve and skin tissues. We focused on the Mo neural-stimulation electrodes in contact with nerve tissue and the SBPUE-encapsulated Mo power-receiver electrodes in contact with skin tissue, following the implantation of the BCC neurostimulator. As shown in fig. S16, no obvious inflammatory infiltration occurred in the BCC group compared to that in the sham group. Immunofluorescence was used to analyze cell population changes and the in vivo immune response in the vagus nerve tissues after 4 weeks of implantation. Neuronal nuclei (NeuN) and S100 calcium binding protein B (S-100β) were chosen to mark neuron and Schwann cell, while TNF-α was chosen to indicate the immune response. The BCC group showed no significant difference in Schwann cell and neuron populations compared to the sham group (Fig. 3C). Similarly, TNF-α expression levels did not differ significantly between the BCC and sham groups (Fig. 3D). The BCC neurostimulator in contact with the skin also did not induce any significant immune or inflammatory response or tissue injury in the surrounding skin tissue (TNF-α indicated inflammation and caspase-3 related to apoptosis and necrosis) (Fig. 3, E and F, and fig. S17). These results suggest that the BCC neurostimulator has good biocompatibility and does not provoke suggestive immune responses in vivo.

The in vitro biodegradability of the components of the BCC neurostimulator, i.e., Mo and SBPUE, was investigated. Weight changes of the Mo and SBPUE films (immersed in 1× PBS at 37°C) showed that they biodegraded slowly (fig. S18A); thus, their shapes remained unchanged during the first month to match the disease treatment period (fig. S18B). Throughout the entire degradation process of the Mo electrode, cracks appeared, fragments fell off, and new cracks were formed (fig. S18C). Consequently, the electrochemical surface area of the electrode initially increased and then decreased. This fluctuation is also the cause of the corresponding rise and subsequent decline in electrochemical performance (Fig. 2H). Moreover, the long-term in vivo implantation process demonstrated that the BCC neurostimulator had good degradability, retained its shape well within 4 weeks, and completed most of the degradation process within 12 weeks (Fig. 3G). This timeline matches the preset disease treatment period and enhances coherence. The entire degradation process had no marked impact on the major organs of the rats (fig. S19).

### Electroceutical treatment of PIBD with the BCC neurostimulator

**Fig. 4.**
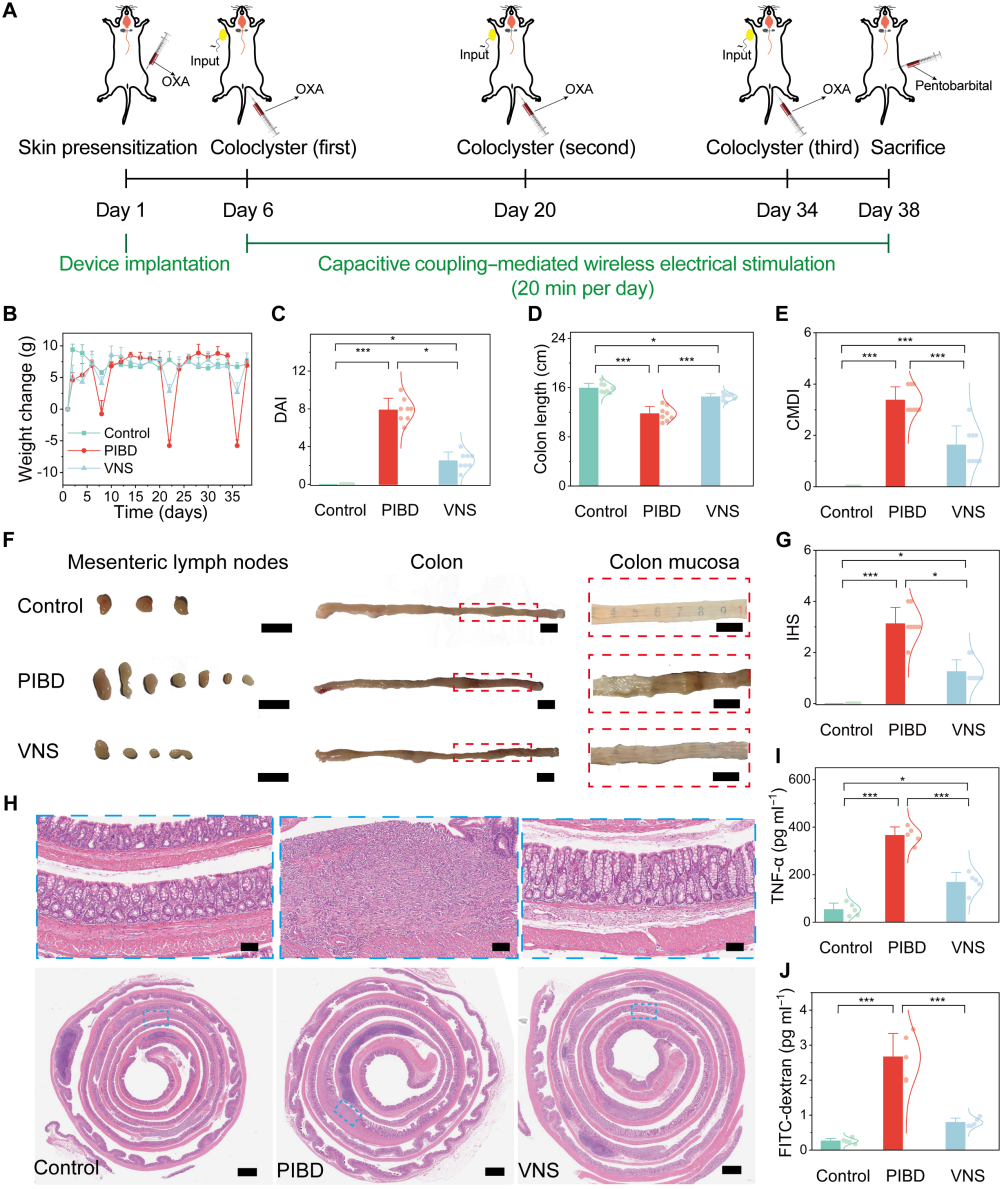
Electroceutical treatment of PIBD with chronic vagus nerve stimulation enabled by the BCC neurostimulator. (**A**) Experimental timeline of capacitive coupling–meditated wireless electrical stimulation in the OXA-induced PIBD model. (**B** and **C**) Average body weight change (B) and the activity index (DAI) score (C) over time in the different groups (*n* = 5). (**D**) Colon length changes in the different groups (*n* = 5). (**E**) Colon mucosal damage index (CMDI) of rats’ colons in the different groups (*n* = 5). (**F**) Representative photographs of mesenteric lymph nodes, colon, and colonic mucosa from rats in the different groups. Scale bars, 10 mm. (**G**) Inflammation-related histology score (IHS) of rats’ colons in the different groups (*n* = 5). (**H**) Representative H&E staining images of rats’ colons in the different groups. Scale bars, 100 μm (top) and 1 mm (bottom). (**I**) Intestinal concentrations of TNF-α in the different groups (*n* = 5). (**J**) Serum concentrations of FITC-dextran in the different groups (*n* = 5). Data are presented as the mean ± SD in (B), (C), (D), (E), (G), (I), and (J) and were analyzed by one-way ANOVA first, and then by the Tukey′s post hoc test. *P ≤ 0.05, ***P ≤ 0.001. NS, not significant.

In vivo experiments were performed to investigate the therapeutic effects of capacitive-coupling wireless electroceutical treatment in a rat model of PIBD. As illustrated in fig. S20, within the same gender cohort, the mortality rate following administration of a 30 mg/kg oxazolone (OXA) enema solution (OXA1 group) exceeded 50%, remarkably surpassing that observed in the normal saline group (NaCl group) and the 10 mg/kg OXA enema group (OXA2 group). The disease activity index (DAI), colon mucosal damage index (CMDI), intestinal inflammatory infiltration, and inflammation-related histological score (IHS) were markedly higher in the OXA1 group when compared to the OXA2 group. At an equivalent dose of OXA enema, male rats exhibited higher mortality rates than females, yet the intestinal pathological damage in surviving males was less severe than that observed in female pups. Thus, considering the survival rate, reliability, and reproducibility of PIBD model construction, the use of 3-week-old female Sprague Dawley rat pups at a 10 mg/kg dose is recommended for subsequent experiments. The rat PIBD model was then induced by multiple OXA enemas (Fig. 4A).

The rats in the VNS group were implanted with the BCC neurostimulator on day 1, and the capacitive-coupling wireless electroceutical treatment (power-transmitter input of ±4 V, pulse width of 0.6 ms per phase, and stimulation frequency of 1 Hz) was administered for 20 min per day. Changes in the body weight of the rats were monitored during the overall process. Because these rats were in the growth and development phase, they experienced a weight decrease in the acute phase (days 1 to 3 after the OXA enema) due to intestinal damage caused by OXA, but their weight rebounded during the remission phase. The weight loss in the PIBD group in the acute phase was much higher than that in the control group, while the capacitive-coupling wireless electroceutical treatment relieved the weight loss in the VNS group (Fig. 4B and fig. S21). DAI score was assessed based on body weight, fecal traits, and fecal occult blood (*39*). As shown in Fig. 4C, the DAI of the PIBD group was significantly higher than that of the control group owing to weight loss, watery diarrhea, and bloody stools, while capacitive-coupling wireless electroceutical treatment alleviated these symptoms. Compared to the control group, the PIBD group exhibited a shortened colon and swollen mesenteric lymph nodes, which appeared as bead-like enlargement (Fig. 4D). The CMDI was significantly elevated in the PIBD group (Fig. 4E), featuring adhesion between the colon and small intestine, severe colonic congestion and edema, intestinal wall thickening and stiffness, and full-thickness necrosis in the distal colon with visible ulcers (Fig. 4F). Compared to the PIBD group, these symptoms were relieved in the VNS group.

The IHS was significantly elevated in the PIBD group but decreased in the VNS group (Fig. 4G). There was a visible inflammatory cell infiltration in the PIBD group (Fig. 4H). While low-level inflammation and scattered immune cell infiltration were observed in the proximal colon segments in the PIBD group, the mid- and distal segments showed marked morphological disruption. In these areas, the colon structure was disrupted, with crypt loss, transmural inflammation, and notable loss of goblet cells. In contrast, the proximal and mid segments of the colons in the VNS group showed clearer structure and crypt morphology with scattered immune cell infiltration, and the distal segment also showed less severe inflammation. In addition, TNF-α level in colon tissues increased in the PIBD group but was down-regulated in the VNS group (Fig. 4I). The serum concentration of fluorescein isothiocyanate–dextran, an indicator of intestinal permeability, demonstrated that PIBD increased permeability (Fig. 4J), which was effectively reduced by capacitive-coupling wireless electroceutical treatment. These results indicate that capacitive-coupling wireless electroceutical treatment may alleviate the inflammatory response during PIBD.

### Intestinal immune balance during electroceutical treatment of PIBD

**Fig. 5.**
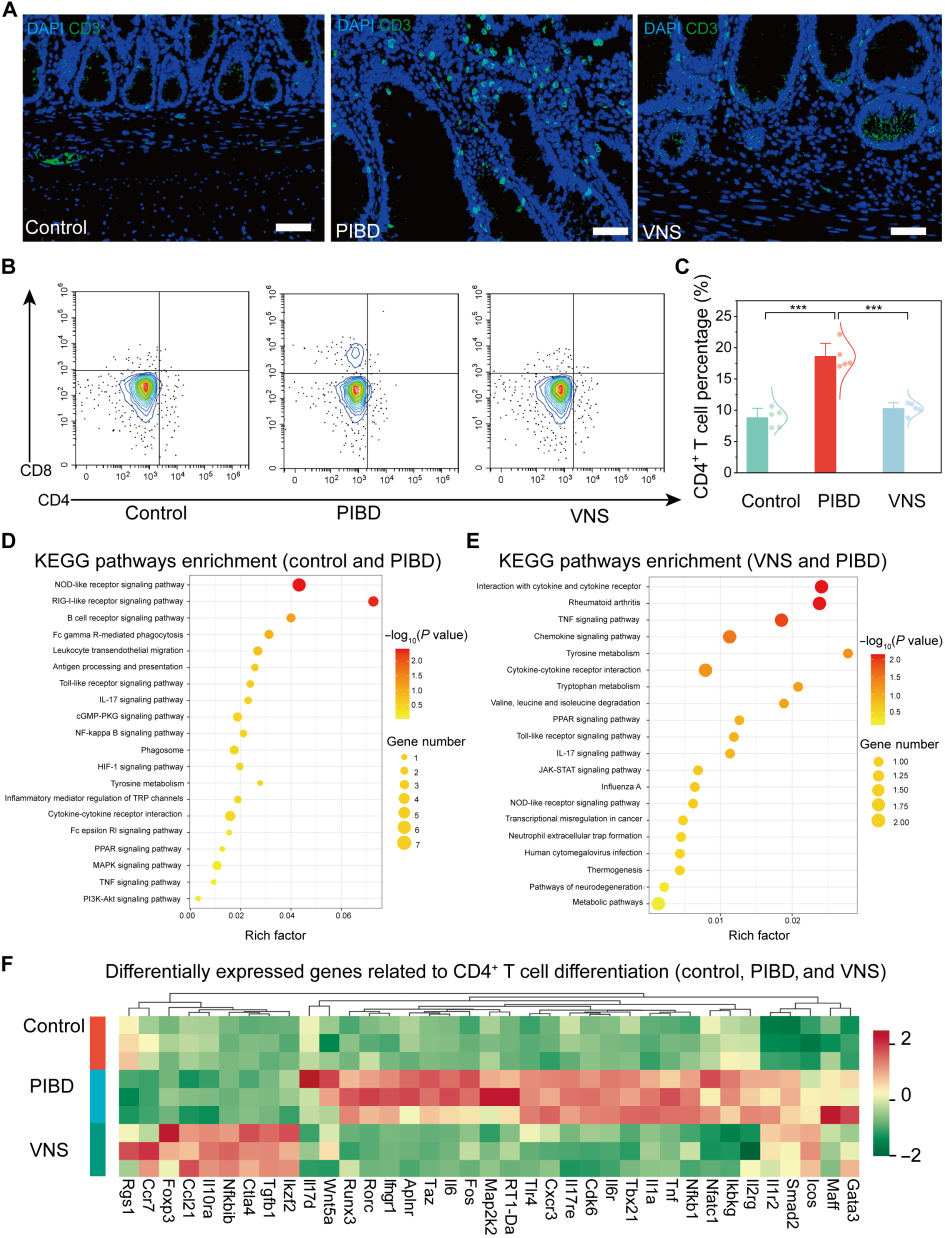
Regulation of gut immunity by BCC neurostimulator-enabled electroceutical treatment in PIBD. (**A**) Representative immunofluorescence staining of T cells in the colon from different groups. Scale bars, 50 μm. (**B** and **C**) FACS plots (B) and statistical analysis (C) of changes in intestinal T cell subtypes from different groups (*n* = 5). (**D**) Kyoto Encyclopedia of Genes and Genomes (KEGG) pathway enrichment bubble plot of the gene expressions (control and PIBD). (**E**) KEGG pathway enrichment bubble plot of the gene expressions (VNS and PIBD). (**F**) Hierarchical clustering heatmap of differentially expressed genes related to the differentiation of CD4^+^ T cells. Data are presented as the mean ± SD in (C) and were analyzed by one-way ANOVA first, and then by the Tukey′s post hoc test. ***P ≤ 0.001. NS, not significant.

Immunofluorescence was used to analyze changes in immune cell populations within the colon. CD3, CD19, NKp46, CD68, CD103, and MPO were used to label T cells, B cells, NK cells, macrophages, dendritic cells, and neutrophils, respectively (*40*, *41*) (Fig. 5A and fig. S22). In the control group, there was minimal infiltration of adaptive immune cells such as T and B cells (fig. S23A). Innate immune cells, including macrophages and neutrophils, were mainly scattered in the submucosal and muscle layers (fig. S23B). In contrast, the PIBD group showed lymphoid follicles in the lamina propria that were primarily composed of T and B cells. In addition, NK cells, macrophages, and neutrophils were present across the lamina propria, submucosa, and muscle layers, with dendritic cells distributed throughout. Notably, T cells infiltrated the colonic mucosa and submucosa of PIBD rats, highlighting them as the primary responsive cells in PIBD. Following capacitive-coupling wireless electroceutical treatment, immune cell infiltration was markedly reduced across all the colon layers.

To elucidate how capacitive-coupling wireless electroceutical treatment regulates the immune microenvironment in PIBD, immune cell species in colon tissues were further investigated using flow cytometry. CD45 (leukocyte common antigen) was used as a molecular marker to identify and isolate immune cells from nonimmune cells (fig. S24A). Within the CD45^+^ population, T cell differentiation was assessed by focusing on CD4^+^ and CD8^+^ T cells. CD4^+^ T cells primarily support immune responses by aiding other immune cells, whereas CD8^+^ T cells act as cytotoxic cells that directly target and kill infected cells. The results showed that PIBD significantly increased the proportion of CD4^+^ and CD8^+^ T cells within the intestinal immune cell population. In contrast, electroceutical treatment significantly reduced the proportion of these cells (Fig. 5, B and C, and fig. S24, B and C).

We investigated the effect of PIBD on immune organs, including the thymus, spleen, and mesenteric lymph nodes. PIBD caused cortical thickening of the thymus, expansion of the white pulp area of the spleen, and enlargement of mesenteric lymph nodes, while electroceutical treatment partially alleviated these symptoms (fig. S25). We also traced the sources of CD4^+^ and CD8^+^ T cells using flow cytometry (fig. S26A). In the thymus, PIBD led to an increase in both CD4^+^ and CD8^+^ T cell proportions, whereas electroceutical treatment reduced these proportions (fig. S26B). In the spleen and mesenteric lymph nodes, PIBD significantly increased the proportion of CD4^+^ T cells but had a minimal impact on CD8^+^ T cell proportions. Electroceutical treatment effectively reduced the proportion of CD4^+^ T cells in these organs with a minimal effect on CD8^+^ T cells (fig. S26, C and D).

To further investigate the mechanism by which the vagus nerve regulates intestinal immunity, we performed high-throughput RNA sequencing of intestinal tissues. The sequencing data from three biological replicates per group, totaling nine samples, were included in the bioinformatics analysis (fig. S27, A to C). Volcano plots highlighted differences in gene expression between the PIBD and control group, as well as between the VNS and PIBD group, identifying up- or down-regulated genes (fig. S27, D and E). In the PIBD group, differentially expressed genes involved in receptor recognition, cytokine production, and immune regulation, such as *Tlr4*, *Tbx21*, and *Il6*, were up-regulated compared to the controls. Conversely, genes encoding antibacterial and antiviral proteins, such as *Oas2* and *Irf7*, were down-regulated. Following electroceutical treatment, anti-infection genes such as *Il10ra* and *Foxp3* were up-regulated, whereas genes promoting immune response and metabolism, including *Il17d* and *Map3k7*, were down-regulated. In addition, pro-inflammatory signaling pathways, such as interleukin-17 (IL-17), nuclear factor κB, and TNF, were up-regulated in the PIBD colon (Fig. 5D). Electroceutical treatment suppressed these pathways, including mitogen-activated protein kinase (MAPK), which may effectively inhibit pro-inflammatory processes (Fig. 5E).

Differentially expressed genes revealed distinct expression patterns in genes related to T cell activation, proliferation, and differentiation across groups, notably in the Notch, Janus kinase-signal transducer and activator of transcription (JAK-STAT), and MAPK families (Fig. 5F) (*42*–*44*). Specifically, T helper 1 (T_H_1) and T helper 17 (T_H_17) cell transcription factors, such as *Tbx21* and *Rorc* were elevated in PIBD but decreased following electroceutical treatment. Conversely, the regulatory T (T_reg_) cells’ specific transcription factor *Foxp3* increased after electroceutical treatment. In T_H_1 cells, IL-12 activates JAK2 and TYK2, leading to STAT4 activation and IFN-γ production, which stimulate T-bet expression (*45*). T_H_17 cells, through STAT3 activation, produce IL-17 and other cytokines, boosting T_H_1 and NK cell functions (*46*). In addition, TGF-β and IL-2 promote T_reg_ development and function via STAT5 activation, leading to FOXP3 expression and IL-10 secretion, thereby contributing to immunosuppression and tissue repair (*47*).

**Fig. 6.**
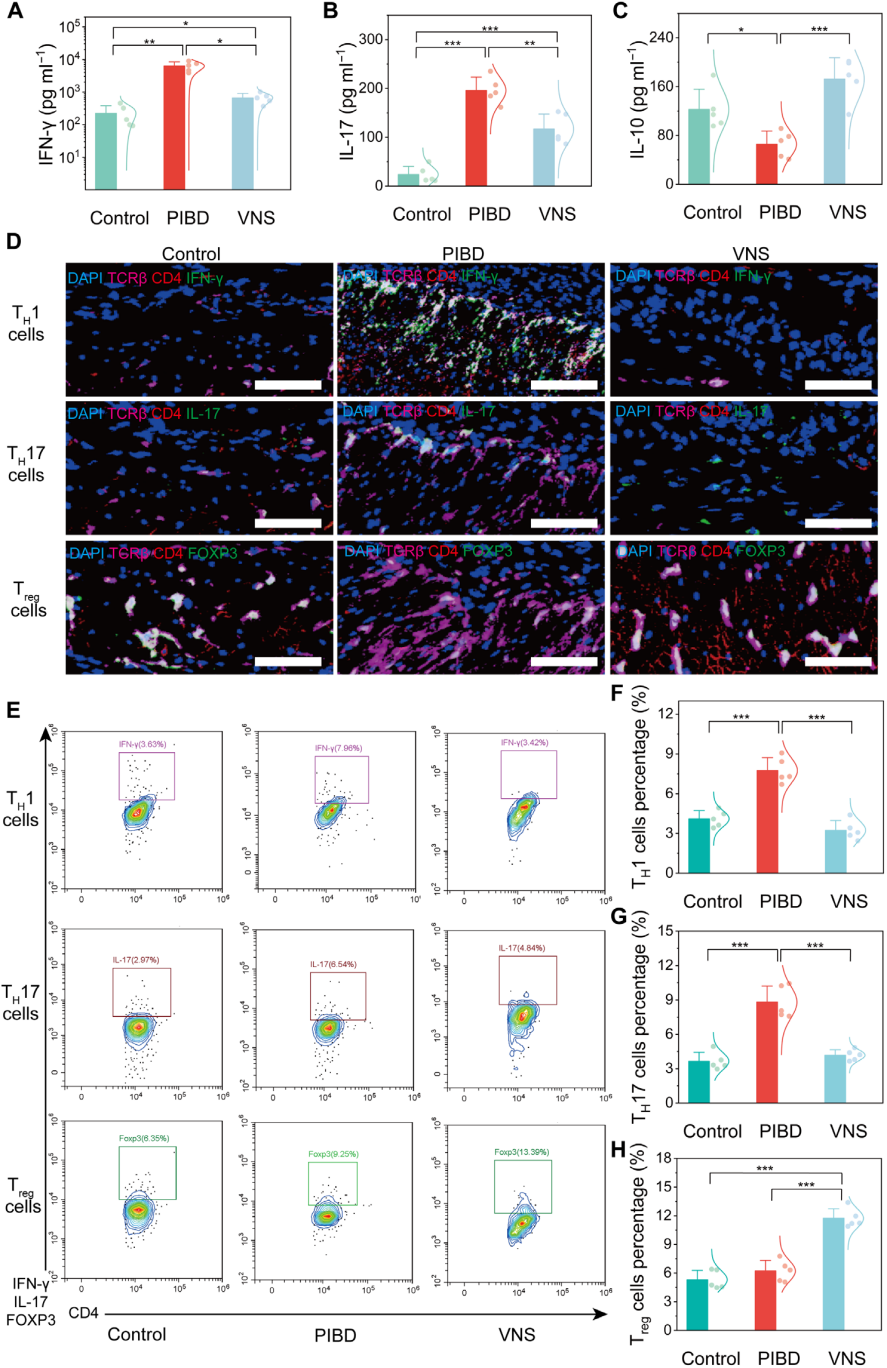
Regulation of intestinal CD4^+^ T cell balance by BCC neurostimulator-enabled electroceutical treatment in PIBD. (**A** and **B**) Concentrations of pro-inflammatory cytokines IFN-γ (A) and IL-17 (B) in intestinal tissue from different groups (*n* = 5). (**C**) Concentration of the anti-inflammatory cytokine IL-10 in the intestinal tissue from different groups (*n* = 5). (**D**) Representative immunofluorescence staining of T_H_1, T_H_17, and T_reg_ cells in the colon from different groups. Scale bars, 50 μm. (**E** to **H**) FACS plots and statistical analysis of changes in intestinal CD4^+^ T cell subtypes in different groups (*n* = 5). Data are presented as the mean ± SD in (A), (B), (C), (F), (G), and (H) and were analyzed by one-way ANOVA first, and then by the Tukey′s post hoc test. *P ≤ 0.05, **P ≤ 0.01, ***P ≤ 0.001. NS, not significant.

Given the role of CD4^+^ T cell differentiation in PIBD development, we investigated the effect of electroceutical treatment on this process. Pro- and anti-inflammatory cytokine secretions by T cells during treatment were analyzed by enzyme-linked immunosorbent assay and reverse transcription quantitative polymerase chain reaction (RT-qPCR), respectively. The concentrations and relative mRNA expression levels of pro-inflammatory cytokines (TNF-α, IFN-γ, IL-1β, IL-6, and IL-17) in the colons of the PIBD group were significantly elevated, while these indicators were down-regulated in the colons of the VNS group (Fig. 6, A and B, and figs. S28 and S29). Conversely, the concentration and relative mRNA expression levels of anti-inflammatory cytokines (IL-10) in the colon of the VNS group were significantly up-regulated (Fig. 6C and fig. S29). These pro-inflammatory cytokines are associated with T_H_1 and T_H_17 cells, while anti-inflammatory cytokine IL-10 is linked to T_reg_. Infiltration of these cells into the intestinal tissue was visually confirmed by immunofluorescence analysis (Fig. 6D and fig. S30). Specifically, CD4^+^TCRβ^+^IFN-γ^+^ T_H_1, CD4^+^TCRβ^+^IL-17^+^ T_H_17, and CD4^+^TCRβ^+^FOXP3^+^ T_reg_ cells were rarely found in the colons of the PIBD group, whereas the numbers of T_H_1 and T_H_17 cells notably increased and were widely distributed. After electroceutical treatment, the numbers of CD4^+^ T cells, T_H_1, and T_H_17 cells decreased, whereas T_reg_ cells increased notably and became widely distributed in the colons of VNS group.

The effect of electroceutical treatment on the balance between pro-inflammatory and anti-inflammatory effects in intestinal tissue was quantitatively analyzed using flow cytometry (Fig. 6E and fig. S31). PIBD led to a remarkable increase in the proportion of CD45^+^CD4^+^TCRβ^+^IFN-γ^+^ T_H_1 and CD45^+^CD4^+^TCRβ^+^IL-17^+^ T_H_17 cells, while electroceutical treatment reduced their proportion and increased the proportion of CD45^+^CD4^+^TCRβ^+^FOXP3^+^ T_reg_ cells (Fig. 6, F to H). In addition, flow cytometry analysis revealed the same phenomenon in primary immune organs related to T cells in the thymus (fig. S32), spleen (fig. S33), and mesenteric lymph nodes (fig. S34). These results suggest that electroceutical treatment regulates and rebalances the proportion of inflammatory and anti-inflammatory CD4^+^ T cells in primary immune organs, collectively modulating the intestinal immune environment.

## DISCUSSION

To address the challenge of constructing neural interface with high biocompatibility and accommodate the dynamic needs of children’s growth and development, it is essential to develop biodegradable electroceutical devices for peripheral nerves with neural electrode fixation approach avoiding stress compression. Herein, we developed a miniaturized, biodegradable, wireless-powered, battery-free BCC neurostimulator that demonstrates efficacy in PIBD treatment. The capacitive-coupling wireless configuration of the BCC neurostimulator enables efficient chronic biphasic electrical stimulation without the need for complex circuitry. It was demonstrated that the self-healing and biodegradable BCC neurostimulator provides stable nerve stimulation and degrades safely posttreatment to minimize tissue damage and immune response. In the rat PIBD model, the BCC neurostimulator alleviated PIBD-related symptoms, including weight loss, colon shortening, increased intestinal permeability, and mesenteric lymph node enlargement. It was further elucidated that the underlying mechanism involves the regulation of anti-inflammatory and pro-inflammatory effects in the intestines by restoring the balance of CD4^+^ T cell subtypes, specifically T_H_1, T_H_17, and T_reg_ cells. Our creation of the BCC neurostimulator suggests a feasible route toward self-powered biodegradable medical implants. Although demonstrated in the rat PIBD model, the BCC stimulator, as a unique bioelectronic medicine, holds great promise in various refractory inflammatory diseases, including rheumatoid arthritis septicemia, and post–myocardial infarction inflammation.

## MATERIALS AND METHODS

### Preparation of BCC neurostimulator

A biodegradable and self-healing elastomer (SBPUE) was used to encapsulate the BCC neurostimulator, preventing it from direct contact with the surrounding biofluids. The typical polymerization procedure for SBPUE is described below. PCL (14.5 g, 7.25 mmol) and poly(tetramethylene ether glycol) (PTMEG) (7.25 g, 7.25 mmol) were mixed in a dried glass flask equipped with a mechanical stirrer. The mixture was heated at 100°C under vacuum for 1 hour to remove moisture and then cooled to 70°C. Isophorone diisocyanate (6.77 g, 30.45 mmol) and dibutyltin dilaurate (DBTDL) (0.050 g, 2000 parts per million) dissolved in dimethylacetamide (DMAc) (5 ml) were added dropwise to the flask and stirred for 2 hours under a nitrogen atmosphere. After synthesizing the prepolymer, the flask was cooled to room temperature. Dimethylglyoxime (1.683 g, 14.50 mmol) dissolved in DMAc (10 ml) was added as a chain extender. The flask was sealed, and the reaction proceeded for another 12 hours. Finally, the concentration of the SBPUE solution was adjusted to 30 wt % by adding DMAc (30 ml) for further characterization.

Neurostimulation electrodes, device wiring, and power receiver electrodes for the BCC neurostimulator were designed using AutoCAD software and fabricated from laser-cut Mo strips (20 μm thick). The power-receiver and grounding electrodes were circular with diameters of 10 and 5 mm, respectively. They were connected to the neurostimulation electrodes through snake-shaped Mo wires that were 10 mm long and 0.2 mm wide. The two neurostimulation electrodes were 0.1 mm wide and 5 mm long, with a 1-mm gap between them. The drawn patterns on the Mo foil were cut using a femtosecond laser (Axinite IR30, Suzhou Bailing Laser Co. Ltd.). The laser parameters were set to a repetition rate of 1 kHz and a power of 1.5 W. The cut Mo strips were placed on a tacky, partially cured SBPUE substrate. Additional uncured SBPUE coatings ensured strong adhesion, thereby forming a top encapsulating layer. The power-receiver electrode was fully encapsulated, whereas only one side of the grounding electrode was covered.

### Construction of the capacitive-coupling system

The entire capacitive-coupling system was constructed using internal (BCC neurostimulator) and external wireless-powering components. The external part used a 10-mm-diameter circular Au foil (encapsulated on both sides) as the power-transmitter electrode and a small rectangular Mo sheet (encapsulated on one side) as the external grounding electrode. They were connected to the signal input terminal by enameled copper wires with a diameter of 40 μm. ArbExpress software (Tektronix, USA) was used for waveform editing, generating a composite waveform consisting of a sine wave (with a frequency of 1 MHz and an amplitude of 0.4 V) superimposed with a trapezoidal wave. The edited waveform was imported into a function generator (AFG3021C, Tektronix, USA), connected to a broadband amplifier (ATA-1200B, Aigtek, China). The broadband amplifier outputs a signal to the external wireless-powering part.

### Construction of the PIBD model

Animal experiments were conducted in accordance with the guidelines approved by the Institutional Animal Care and Use Committee of Huazhong University of Science and Technology (TJH-202109021). The 3-week-old specific pathogen-free (SPF) Sprague Dawley juvenile female rats were housed in the barrier system at Laboratory Animal Center of Tongji Hospital affiliated to Tongji Medical College of Huazhong University of Science and Technology. The feed and purified water were regularly replaced, and the housing environment adhered to the national standard Laboratory Animal-Requirements of Environment and Housing Facilities (GB14925-2010). Animals were housed individually in adaptive feeding for 3 days before surgery.

The PIBD modeling process involved a series of experimental procedures in juvenile rats. First, skin sensitization treatment was applied by spreading OXA-sensitizing solution on hairless areas of the backs of rats. Subsequently, on days 6, 20, and 34, OXA was administered via enema at varying doses. Throughout the experiment, the rats’ weights, stool characteristics, and other health indicators were monitored regularly. Finally, on day 38, dissection was conducted to observe the general condition of the colon and mesenteric lymph nodes, as well as microscopic changes in the mucosa.

### Long-term VNS by BCC neurostimulator

In the VNS group, the left cervical vagus nerve of anesthetized rats was dissected for BCC neurostimulator implantation. Biphasic, charge-balanced rectangular current pulses (a power-transmitter input of ±4 V, a pulse width of 0.6 ms per phase, and a stimulation frequency of 1 Hz) were applied to stimulate the vagus nerve 20 min daily for 4 weeks. During stimulation, the rats were anesthetized with isoflurane using an animal research anesthesia machine (R583S, RWD).

### Statistical analysis

Statistical analysis was conducted with Origin 2023 software by analysis of variance (ANOVA) and followed by the Tukey’s post hoc test. Data are presented as mean ± SD. The significance threshold is presented as *P ≤ 0.05, **P ≤ 0.01, and ***P ≤ 0.001. NS, not significant.
