# Supplementary material for: A biodegradable capacitive-coupling neurostimulator for wireless electroceutical treatment of inflammatory bowel diseases
Source: Sci Adv. 2025 Feb 14;11(7):eadu5887. doi: 10.1126/sciadv.adu5887 (PMC11827631; doi:10.1126/sciadv.adu5887)
Supplement: Supplementary file 1 — Supplementary Text Figs. S1 to S34 Table S1 Legend for movie S1 [file sciadv.adu5887_sm.pdf]

Science Advances
AAAS

Supplementary Materials for

A biodegradable capacitive-coupling neurostimulator for wireless electroceutical treatment of inflammatory bowel diseases

Qiong Wang *et al.*

Corresponding author: Jiexiong Feng, 2002tj0515@hust.edu.cn; Cunjiang Yu, cunjiang@illinois.edu; Zhiqiang Luo, zhiqiangluo@hust.edu.cn

Sci. Adv. **11**, eadu5887 (2025)
DOI: 10.1126/sciadv.adu5887

The PDF file includes:

Supplementary Text
Figs. S1 to S34
Table S1
Legend for movie S1

Other Supplementary Material for this manuscript includes the following:

Movie S1

Supplementary Text

Materials

Polycaprolactone diol (PCL) (Mw = 2000), poly(tetrahydrofuran) (PTMEG) (Mw = 1000), isophorone diisocyanate (mixture of isomers, 99%), dibutyltin dilaurate (DBTDL, 95%), N-N-Dimethylacetamide (DMAc, ≥ 99.9%), dimethylglyoxime (98%), and Calcein AM (≥ 96%) were purchased from Aladdin. Lipase (porcine pancreas) was purchased from Shanghai Pureone. 1× PBS (0.01 M, pH = 7.2~7.4) was purchased from Beijing Chreagen. The Cell Counting Kit-8 (CCK-8) and propidium iodide (PI, ≥ 94%) were purchased from Beyotime. Medium with high glucose (DMEM), penicillin-streptomycin solution (X100), and fetal bovine serum were purchased from Hyclone. Molybdenum foil (thickness, 20 μm) was purchased from Haoxuan Metal Materials. The DI water was collected using a Milli-Q water purification system (Millipore). All reagents were used as received, without purification. FITC-dextran, oxazolone (OXA) and olive oil were purchased from Sigma-Aldrich. Anti-CD103 antibody was purchased from Beijing Bioss. Anti-CD19 antibody, anti-CD3 antibody, anti-IFN-γ antibody, and anti-IL-17A antibodies were purchased from Wuhan Proteintech. Anti-NKp46 antibody was purchased from BOSTER. Anti-CD68 antibody and anti-FOXP3 antibody were purchased from ABclonal. Anti-MPO antibody was purchased from Abcam. Anti-TCRβ antibody was purchased from Beyotime. Anti-CD4 antibody was purchased from NOVUS. EDTA (5 mM), HEPES (10 mM), collagenase D, trizol, and monoclonal antibodies (TCRβ, FOXP3, IFN-γ, and IL-17A) were purchased from Thermo Fisher. DNase I was purchased from STEMCELL Technologies. Dispase II was purchased from Roche. Monoclonal antibodies (CD45, CD8, and CD4), fixable viability stain 510 (FVS510), purified mouse anti-rat CD32, transcription factor buffer, set and leukocyte activation cocktail were purchased from BD Biosciences.

Chemical structure characterization

The chemical structure of SBPUE was studied using nuclear magnetic resonance spectroscopy (NMR, AV400, Bruker, Switzerland) and Fourier transform infrared spectroscopy (FTIR, Nicolet iS50R, USA).

Electrochemical characterization

A three-electrode system was used to characterize the electrochemical properties of Mo neurostimulation electrodes in 1× PBS. This setup included a working electrode (Mo electrode), a counter electrode (1 × 1 cm Pt sheet), and an Ag/AgCl reference electrode. For electrochemical impedance measurements, the frequency range spanned from 10^{-1} to 10^{5} Hz, with a 5-mV amplitude sine wave current and a 0-V direct current potential. And the charge injection measurement was conducted with biphasic pulses of 1.5 ms and ± 0.5 V in 1× PBS. The charge injection density (Q_{inj}) was calculated using Equation (1):

$$Q_{inj} = \frac{Q_c + Q_a}{A} \tag{1}$$

Where Q_c represents the amount of charge through the cathode, Q_a represents the amount of charge through the anode, and A represents the area of Mo electrode.

To measure the charge storage capacity (CSC), the cyclic voltammetry was applied with the scanning potential between -0.5 and 0.5 V, and a scan rate of 100 mV s^{-1}. CSC was calculated from the measured CV data using Equation (2):

$$CSC = \int_{E2}^{E1} \frac{i(E)}{2vA} dE \quad (2)$$

Where *v* is the scan rate, *E1* and *E2* are the potential windows, *i* is the current at each potential, and *A* is the electrode area.

To assess electrochemical stability, the Mo electrodes were immersed in 1× PBS (37 °C) for 4 weeks. The charge injection, charge storage, and impedance properties of the Mo electrodes were evaluated every week.

To assess electrochemical stability, the Mo electrodes (Mo wires) were immersed in 1× PBS (37 °C) for 4 weeks. The charge injection, charge storage, and impedance properties of the Mo electrodes were evaluated every week. The surface morphology of Mo wires was also characterized weekly using SEM (Hitachi SU8010).

Mechanical and self-healing tests of SBPUE

A 30 wt% DMAc solution of SBPUE was loaded into a square Teflon mold. The mold was gradually heated from 40 to 130 °C for 24 h. The residual solvent was removed by vacuum drying at 60 °C for 12 h until no bubbles were observed in the samples. Mechanical performance was measured using a universal testing machine (CTM8000, Xie Qiang Instrument Manufacturing). For the tensile test, dumbbell-shaped SBPUE samples (30 mm × 5 mm × 2 mm) were used. To measure the tensile properties of SBPUE, a stretching rate of 50 mm·min^{-1} was used. The elastic modulus of the SBPUE was calculated from the slope of the linear section of the tress-strain curve. For the cyclic tension test, SBPUE was stretched to strain of 20%. The tensile testing speed was 20 mm·min^{-1}, the compression speed was 5 mm·min^{-1}, and the number of cycles was 10. The compression test was conducted on a cuboidal (10 mm × 10 mm × 7 mm) specimen. The specimen was compressed at a speed of 2 mm min^{-1} at room temperature (25 °C) at a strain ratio of 50%. The compressive modulus of the SBPUE was calculated from the slope of the linear section of the stress-strain curve.

The shear modulus was tested using a rheometer (MCR102, Anton Paar, Austria) set to operate at 37 °C. Angular frequency sweep measurements were conducted over a range from 0.1 to 10 rad s^{-1} with a 0.5% amplitude. And the shear modulus was calculated using Equation (3):

$$E = 2\sqrt{G'^2 + G''^2} \cdot (1 + v) \quad (3)$$

Where E represents the shear modulus, G' and G" represent the storage and loss moduli at 1 Hz, respectively, and v representing the Poisson's ratio, is assumed to be 0.5. To measure the tensile properties of the standalone serpentine wire, a stretching rate of 1 mm·min^{-1} was used.

The self-healing test primarily assessed the scratch recovery and restoration of mechanical properties at room temperature over various time intervals. Scratch recovery tests were performed by scratching the neat films with an 18G needle. Scratches were crossed over another obliquely to easily track the changes in scratch-width using an optical microscope. The restoration of the mechanical properties was assessed by pressing two SBPUE specimens (15 mm × 5 mm × 2 mm) under a plumb over a specified time at room temperature. The peeling test and the lap shear test

were conducted at speed of 20 mm min^{-1} after the pressing process. For the peeling test, the interfacial toughness was calculated by dividing twice the peeling force by the width of overlapping area. For the lap shear test, the adhesive strength was calculated by dividing the maximum peeling force by the overlapping area.

Cytocompatibility Assay

The cytocompatibility of SBPUE was detected by CCK-8 and live-dead cell staining using L929, PC12, and ND7/23 cells. The BCC neurostimulator was immersed in Dulbecco's modified Eagle medium (DMEM) at 37 °C for 24 h to obtain the extract (100 mg BCC in 1 mL DMEM). The BCC neurostimulator extract was supplemented with 10% v/v fetal bovine serum and 100 U mL^{-1} penicillin-streptomycin before use. L929, PC12, and ND7/23 cells were seeded in 24-well tissue culture plates at a density of 3000, 2000 and 1500 cells/well, respectively. The culture plates were incubated at 37 °C and 5% CO_2 for 1, 3 and 5 days. Cell viability was quantitatively determined using the CCK-8 assay. In addition, cells were seeded in a 6-well tissue culture plate at a density of 10000, 6000, and 6000 per well, and incubated at 37 °C and 5% CO_2 for 1, 3 and 5 days. Cell morphology was visually assessed by live-dead staining.

Measurement of open-circuit voltage and short-circuit current

The external grounding electrode was aligned with the grounding electrode of the BCC neurostimulator, and the power-transmitter electrode was aligned with the power-receiver electrode of the BCC neurostimulator. The power-transmitter electrode was connected to the positive output of the broadband amplifier, and the external grounding electrode was connected to the negative output. The power-receiver electrode of the BCC neurostimulator was connected to the positive probe of a digital oscilloscope, and the grounding electrode to the ground. The input voltage was adjusted from 2.5 V to 5 V by changing the gain of the broadband amplifier, allowing the digital oscilloscope to display the open-circuit voltage of the coupling devices at each input level. To measure the short-circuit current, the output of a current preamplifier (SR570, Stanford, USA) was connected to a digital oscilloscope via a BNC interface. The power-transmitter and the power-receiver electrodes were then connected to the broadband amplifier output and input of the current preamplifier, respectively. The function generator output voltage was adjusted from 2.5 V to 5 V, and the short-circuit current of the coupling devices at different input voltages was displayed using a digital oscilloscope and current preamplifier. The open-circuit voltage of the power-transmitter and power-receiver electrodes with radii of 5 mm, 3 mm, and 10 mm, and a side length of 5 mm, was tested under an input voltage of 4 V. The distance between the power-transmitter and power-receiver electrode varied by inserting tissues of different thicknesses between them. These tissues included 1 mm of rat skin, 2 mm of chest muscle, 3 mm of stacked muscle and skin, 10 mm of gastrocnemius muscle, and 11 mm of stacked gastrocnemius muscle and skin. The voltage transmission efficiencies were calculated using Equation (4):

$$\frac{V_{out}}{V_{in}} \approx \frac{R_S}{R_S + 2Z} \quad (4)$$

Where Z is circuit's impedance and R_S is the shunt resistance. Z and R_S were measured using a digital bridge (TH2830, Changzhou Tonghui Electronics Co., Ltd.). The output voltage was tested when the electrodes were misaligned by 0 mm, 1 mm, 2 mm, 3 mm, 4 mm, and 5 mm under the 4 V voltage conditions. The SBPUE-encapsulated Mo electrodes were immersed in PBS at 37 °C

for 4 weeks and the electrodes were removed every week, and their open-circuit voltage was measured under an input voltage of 4 V.

Impedance matching with load

The coupling capacitance of the entire capacitive-coupling system was measured using a digital bridge (TH2830, Changzhou Tonghui Electronics Co., Ltd.) when the signal frequency was adjusted from 50 Hz to 100 kHz. An allometric function model was then applied to fit the data and calculate reactance at a working frequency of 1 MHz. The appropriate inductance was selected to achieve matching, using the Equation (3):

$$X = -\frac{1}{2\pi f C} = 2\pi f L \tag{5}$$

Where *X* is the reactance, *f* is the working frequency, *C* is the capacitance, and *L* is the inductance.

The impedances of a 1 cm vagus nerve tissue at different frequencies were measured with the digital bridge, and then fitted with an allometric function model and formula (4) to calculate the resistance of a 1 mm vagus nerve tissue at a working frequency of 1 MHz:

$$R = \frac{\rho L}{S} \tag{6}$$

Where *R* is the resistance, *ρ* is the resistivity of the material, *L* is the length of the material, and *S* is the cross-sectional area.

Based on the calculated resistance of the vagus nerve tissue, loading resistors with values ranging from 2 to 20 kΩ were selected for the current measurement. The output current values under different loads were tested with/without the impedance matching.

Measurement of electrophysiological signals

Electrophysiological signal acquisition was performed using a biological signal acquisition and analysis system (BL-420N; Chengdu Tai Meng Software Co., Ltd.). The left sciatic nerve was exposed and the Mo electrode of the BCC neurostimulator was wrapped around the sciatic nerve. The needle electrodes were inserted into the left gastrocnemius muscle and connected to the signal input of the biological signal acquisition and analysis system. The external device of the capacitive-coupling system was attached to the skin using a medical ultrasound coupling gel aligned with the BCC neurostimulator, and an input stimulation signal was applied to the external device. Changes in muscle action potential before and after stimulation were recorded.

The left gastrocnemius tendon was then separated and connected to the tension sensor of the biological signal acquisition and analysis system using sutures. An input stimulation signal was applied to the external device, and the changes in muscle tension before and after stimulation were recorded. Next, the right vagus nerve was exposed and the Mo electrode of the BCC neurostimulator was wrapped around the vagus nerve. An input stimulation signal was applied to the external device and changes in the nerve action potential before and after the stimulus were recorded.

Measurement of intestinal permeability

SD rats were fasted for 6 h, and FITC-dextran (4 kDa) was dissolved in pure water to a concentration of 50 mg mL^{-1}. FITC-dextran solution was administered at a dose of 600 mg kg^{-1}. Blood from the rats was collected into tubes 4 h later and then centrifuged at 3000 rpm for 10 min to obtain the supernatant. The absorption of FITC-dextran was measured at a wavelength of 520 nm.

Transcriptome Sequencing

The intestines were cut 1.5 cm from the anus to the cecum, rinsed with cold PBS, and cleared of blood and tissues. The lumen was flushed and opened along the mesenteric side, and the contents were removed. The colon was segmented into 1 cm pieces, flash-frozen in liquid nitrogen, and stored at -80 °C. The obtained tissues were rapidly homogenized. Total RNA was extracted using Trizol reagent with a target concentration of at least 1 µg. RNA purity was tested with a Nanodrop ND-2000, and RNA integrity was checked using an Agilent Bioanalyzer. mRNA was isolated from 1 µg of RNA using oligo (dT) beads, fragmented, and used to synthesize cDNA. Adapters were attached, and PCR amplification was performed. Quantification was performed with a Qubit 4.0, size was checked with a bioanalyzer, and the library's effective concentration was quantified using qRT-PCR. Libraries were pooled based on their concentration and sequenced on an Illumina NovaSeq 6000 platform.

Flow cytometer

The intestinal tissues were first washed in saline and the clots and adhesions were removed. The intestinal tissues were cut into 0.3 cm segments, pre-digested, and filtered to remove mucus until the segments were clean. The obtained intestinal segments were further chopped, digested, filtered, and rinsed with culture medium to prepare intestinal single-cell suspensions. The organ tissues were chopped, filtered, grinded, centrifuged to remove red cells and resuspended to prepare single-cell suspensions. The intestinal and other organ tissue suspensions were subjected to viability staining with FVS510, FcγII, and FcγIII receptor (FCR) blocking, surface maker (CD45, CD4, and CD8) staining, and resuspended for flow cytometry analysis of $CD4^+$ and $CD8^+$T cells. To detect T_H1, T_H17 and T_{reg} cells, the single cell suspension was incubated for 6 h using a Leukocyte Activation Cocktail in a 37 °C humidified CO_2 incubator prior to staining with FVS510. After surface staining (CD45, CD4, and TCRβ), cells were fixed and permeabilized using the transcription factor buffer set, followed by intracellular staining (IFN-γ, IL-17, and FOXP3).

Histopathological and immunofluorescence examination

At week 4 post-therapy, the nerves, intestinal tissues, skin, hearts, livers, spleens, lungs, and kidneys of the rats were fixed with 4% paraformaldehyde, dehydrated, and embedded in paraffin. These tissues were subsequently sectioned and stained for HE histopathological analysis. Immunofluorescence staining of NeuN, TNF-α, and S100-β was performed to evaluate nerve injury and inflammation at the neural interface. Immunofluorescence staining of Caspase-3 and TNF-α were employed to evaluate skin tissue injury and inflammation at skin interface. CD68, CD103, MPO, CD3, CD19, and NKp46 were chosen as specific markers to assess intestinal inflammation. Specifically, TCRβ and CD4 were chosen to label $CD4^+$ $TCR\beta^+$ T cells, while INF-γ, IL-17, and FOXP3 were chosen to label T_H1, T_H17, and T_{reg} cells, respectively. The ImageJ software was used for analyzing biomarker fluorescence intensity.

RNA Extraction and RT-qPCR

Total RNA was isolated from the colon using Trizol reagent (Invitrogen, USA). The RNA samples' A260/A280 absorbance ratio was measured using a Nanodrop ND-2000 (Thermo Scientific, USA), and the RNA Integrity Number (RIN) was determined using an Agilent Bioanalyzer 4150 (Agilent Technologies, CA, USA). Subsequently, cDNA was synthesized through reverse transcription using HiScript II Q RT SuperMix for qPCR (+gDNA wiper) (Vazyme, R223-01). Quantitative PCR was performed on a real-time fluorescence PCR system (Applied Biosystems) using ChamQ Universal SYBR qPCR Master Mix (Vazyme, Q711-02), with actin as the internal reference gene. The primer sequences for the target genes are listed in Table S1.

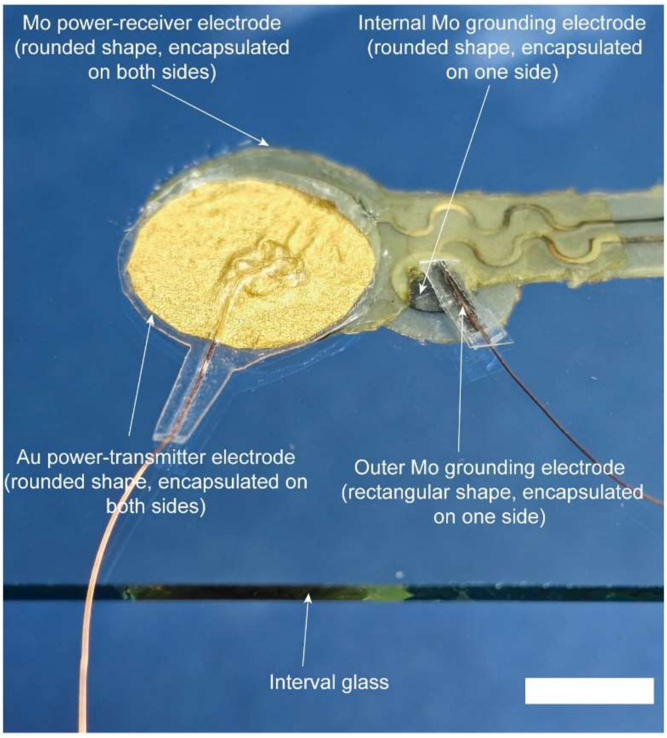

Fig. S1. Photograph of the capacitive-coupling wireless power delivery system. A wearable gold (Au) power-transmitter electrode (rounded shape, encapsulated on both sides) is aligned with the capacitive-coupling Mo power-receiver electrode (rounded shape, encapsulated on both sides). The wearable grounding electrode (rectangular shape, encapsulated on one side) and the implantable grounding Mo electrode (rounded shape, encapsulated on one side) are electrically connected through the skin tissue to ensure stable grounding. Scale bar, 1 cm.

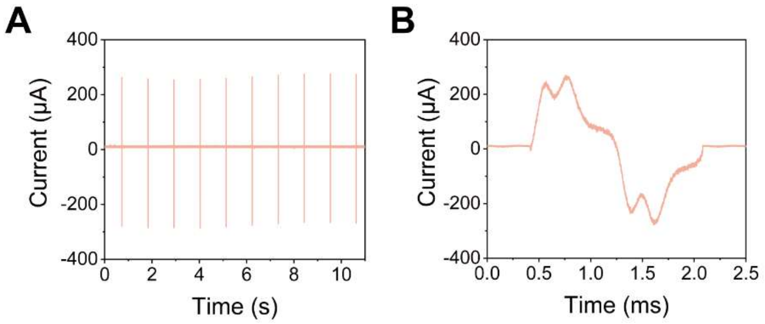

Fig. S2. Short-circuit current generated by capacitive coupling. (**A**) Output current of the Mo power-receiver electrode when a 4 V input voltage is applied at a frequency of 1 Hz. (**B**) Enlarged output current waveform generated by the Mo power-receiver electrode.

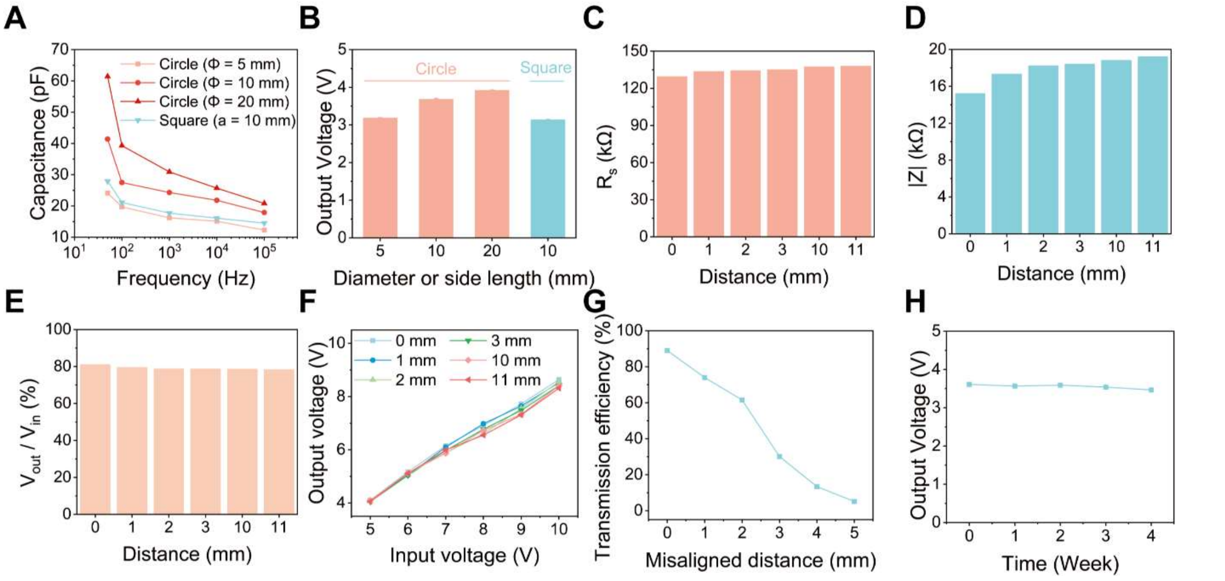

Fig. S3. The effects of electrode shape, size, spacing, misalignment, and degradation on performance. **(A)** Capacitance between electrodes of different sizes and shapes. **(B)** Output voltage of electrodes with different sizes and shapes. **(C)** Impedance of tissues with varied thicknesses. **(D)** System impedance with tissues of different thicknesses. **(E)** Voltage transmission efficiency at different distances. **(F)** Output voltage at different distances. **(G)** Output voltage under electrode misalignment. **(H)** Output voltage during 4-week-degradation.

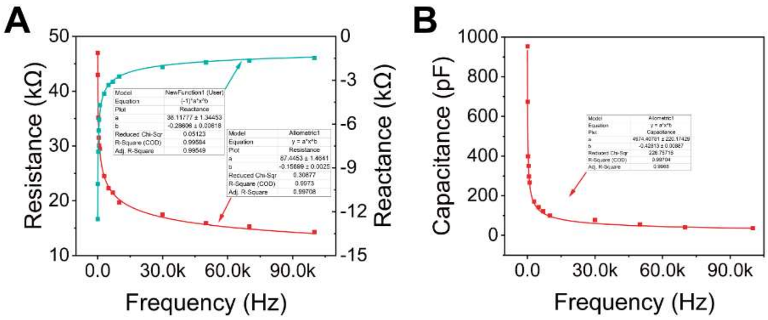

Fig. S4. Measurement and fitting of the resistance, reactance, and capacitance components at varying frequencies. (**A**) Relationships between frequency and resistance (red) or inductive reactance (green) of nerve tissues (1 mm rat vagus nerve). (**B**) Relationship between frequency and capacitance.

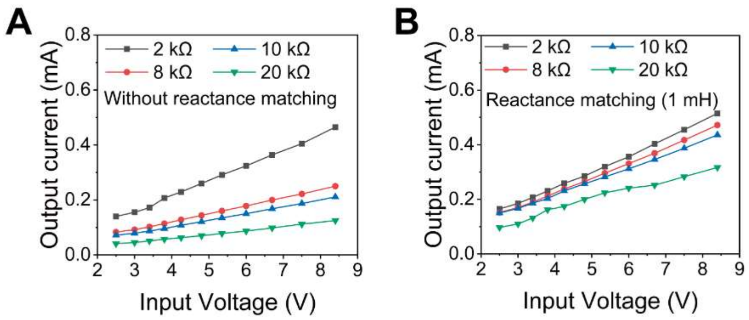

Fig. S5. Dependance of output current on input voltage with different loads. (**A**) Output current of Mo power-receiver electrodes without reactance matching. (**B**) Output current of Mo power-receiver electrodes with reactance matching (adding a 1 mH inductor in the power-transmitter circuit).

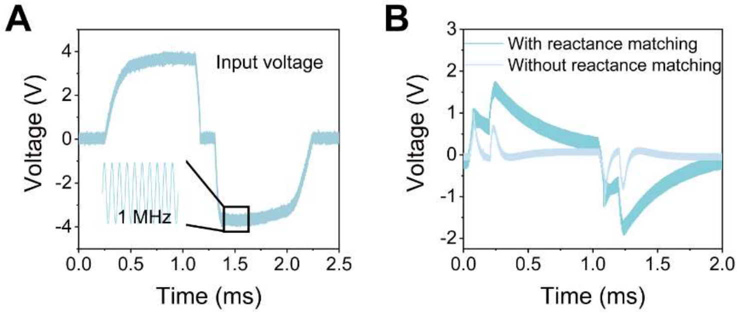

Fig. S6. Voltage response comparison with and without reactance matching under high-frequency capacitive-coupling conditions. (**A**) Designed waveform for the construction of the voltage input. (**B**) Voltage response comparison with and without reactance matching at 1 MHz.

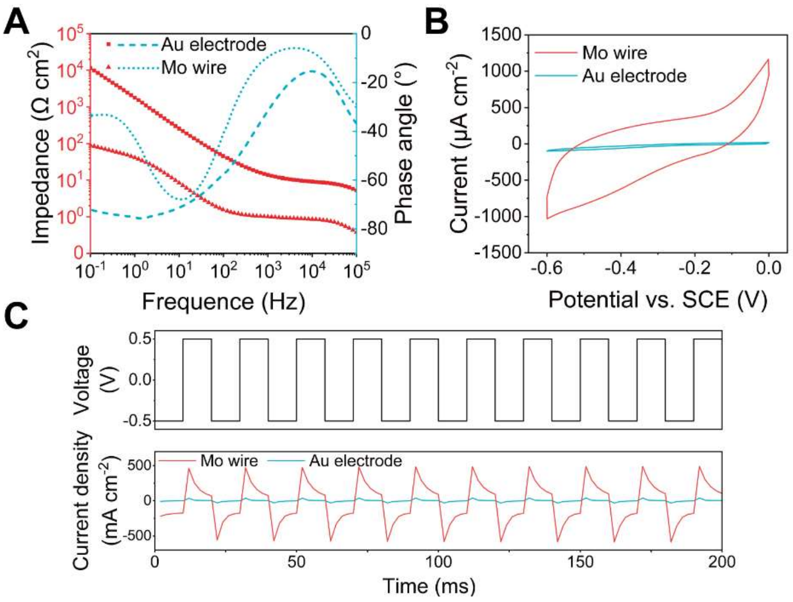

Fig. S7. Electrochemical properties of Mo electrodes. (**A**) Representative bode plots of the Mo and Au electrodes. (**B**) Representative CV curves of the Mo and Au electrodes. (**C**) Representative CIC curves of the Mo and Au electrodes with biphasic pulses (± 0.5 V, 1.5 ms).

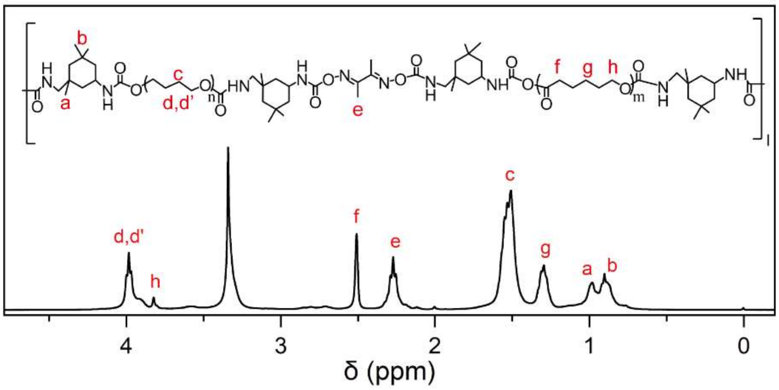

Fig. S8. Structure characterization of SBPUE. The 1H NMR of the developed SBPUE confirms its successful synthesis.

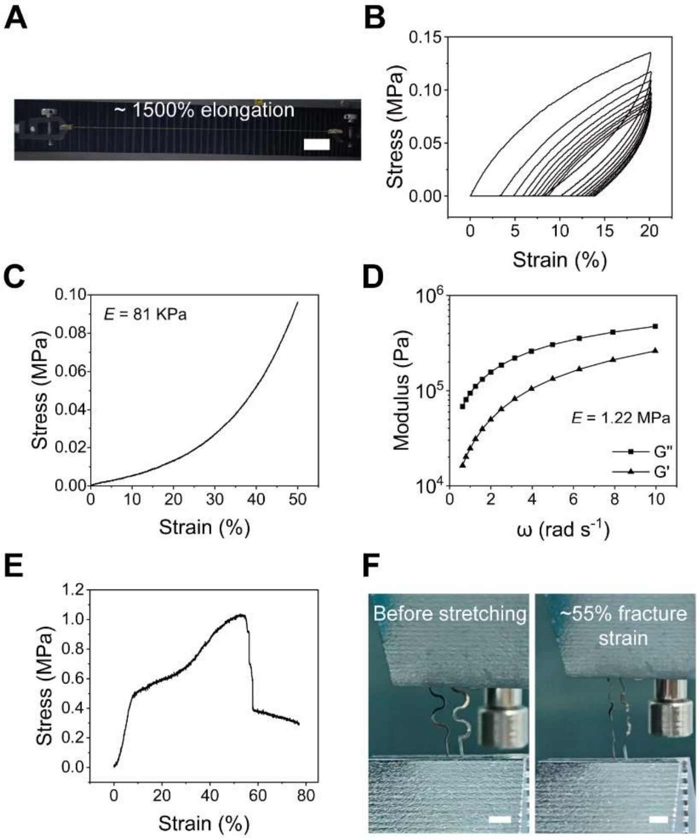

Fig. S9. Mechanical properties of SBPUE. (A) Stretchability of the SBPUE with a linear strain of ~1500%. **(B)** Consecutive cyclic tensile test of the SBPUE at 20% strain shows approximately 15% of the residual strain. **(C)** The compressive stress-strain curve of SBPUE (less than 50% strain) demonstrates that its compressive modulus is about 81 kPa. **(D)** The rheological variation of G′ and G″ for SBPUE reveals a shear modulus of 1.22 MPa. **(E)** Stress-strain curve of the serpentine wire. **(F)** Photographs of the serpentine wire under stretching. Scale bar, 2 mm.

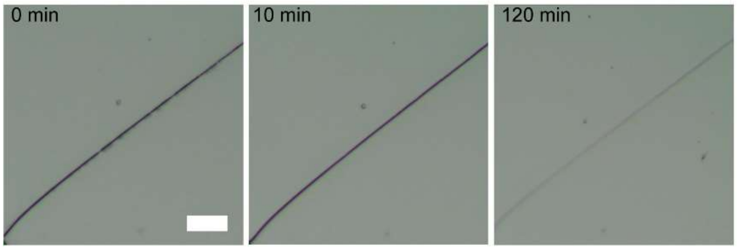

Fig. S10. Optical images of the damaged and healed SBPUE film. It shows the disappearance of the groove after healing at room temperature for 120 min. Scale bar, 100 μm.

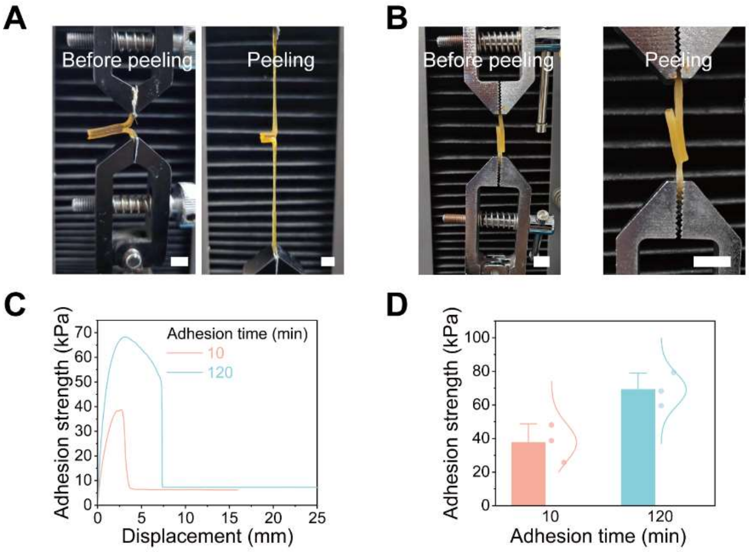

Fig. S11. Self-healing properties of SBPUE. (**A**) Images of peeling test of self-healed SBPUE. Two SBPUE films were self-healed for 120 min. Scale bar, 1 cm. (**B**) Images of lap shear test of self-healed SBPUE. Two SBPUE films were self-healed for 120 min. Scale bar, 1 cm. (**C**) Representative adhesion strength-displacement curves of two self-healed SBPUE films. (**D**) Adhesion strength of self-healed SBPUE (n=3).

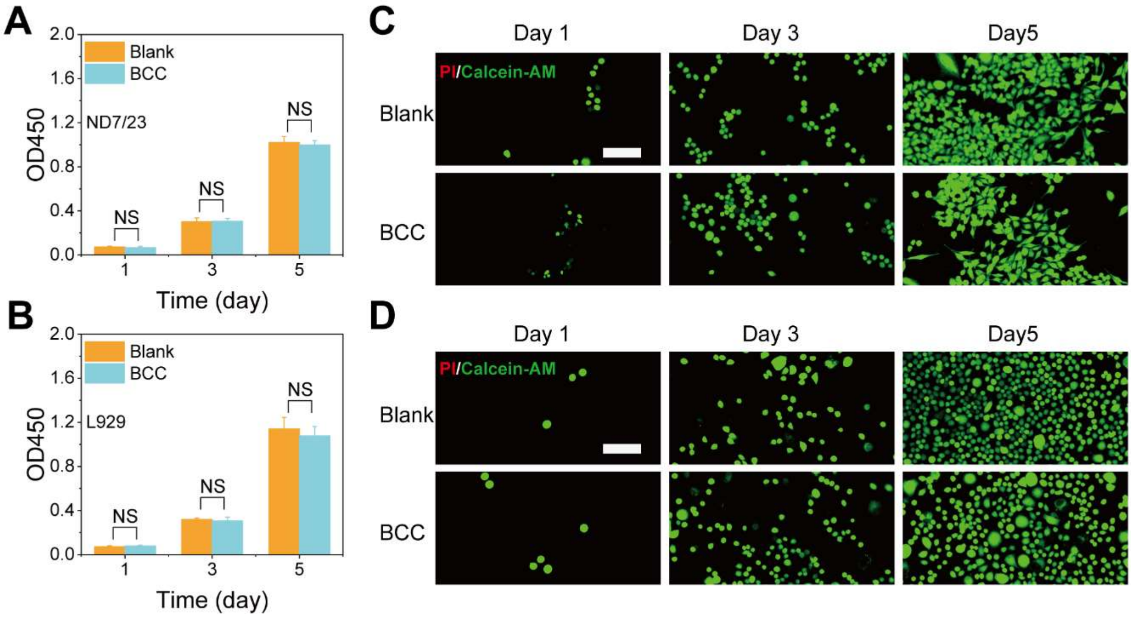

Fig. S12. *In vitro* cytotoxicity assay of BCC neurostimulator. (**A**, **B**) Cell viability of ND7/23 (**A**) and L929 (**B**) cells with the BCC neurostimulator extracts (n = 5). (**C**, **D**) Live-dead staining of ND7/23 (**C**) and L929 (**D**) cells with the BCC neurostimulator extracts. Scale bar, 100 μm. Data are presented as the mean ± standard deviation in (**A** and **B**) and were analyzed by one-way ANOVA first, and then by the Tukey′s post hoc test. NS, not significant.

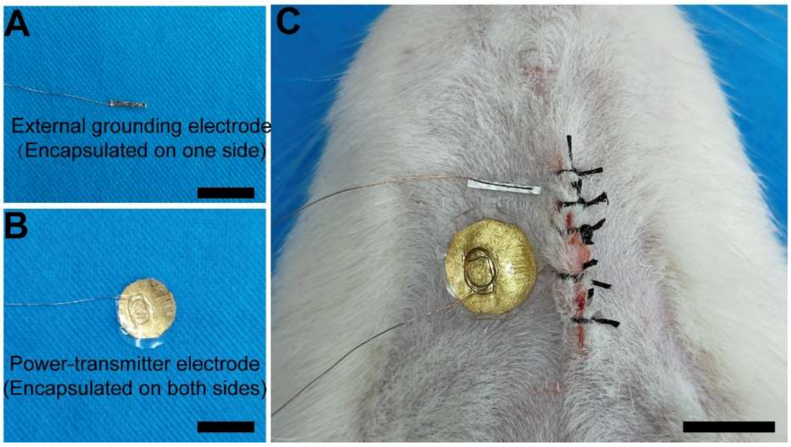

Fig. S13. Photograph of the *in vivo* wireless stimulation system. (**A**) External grounding electrode consists of rectangular Mo encapsulated with PU on one side. (**B**) Power-transmitter electrode consists of Au foil encapsulated with PU on both sides. (**C**) Image of the external wireless stimulation system placement, showing the external and implanted grounding electrodes connected through skin tissue, with the power-transmitter electrode aligned to the implanted power-receiver. Scale bar, 1 cm.

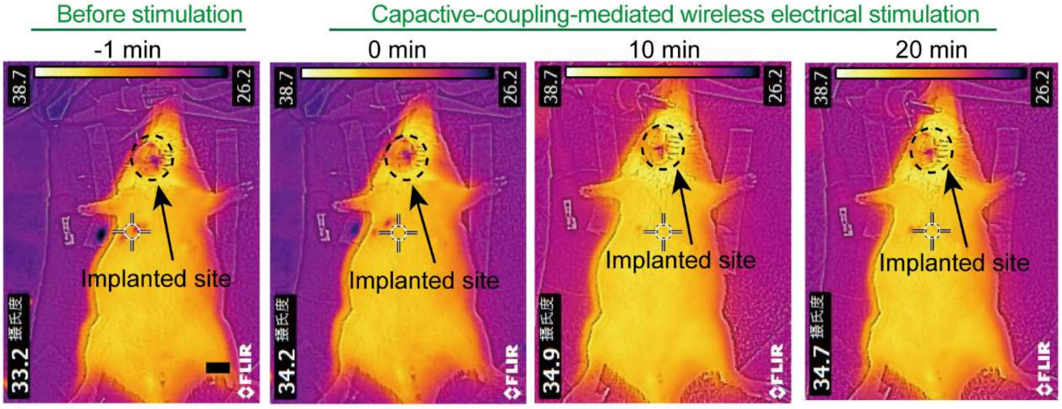

Fig. S14. Temperature variation caused by capacitive coupling. The 20-minute capacitive-coupling stimulation shows insignificant temperature elevating. Scale bar, 1 cm.

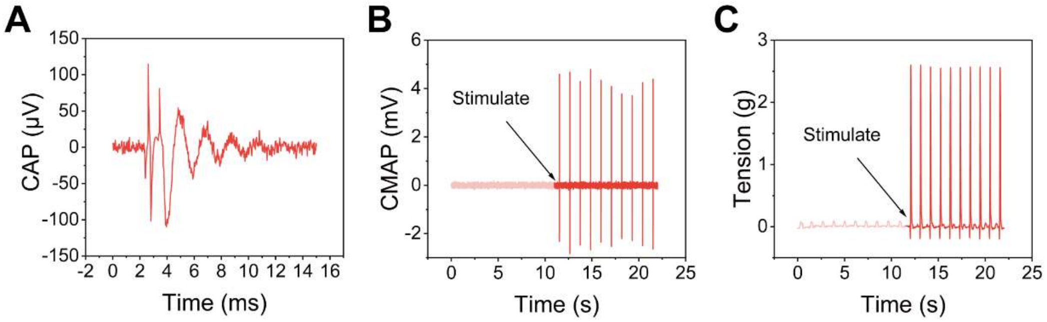

Fig. S15. Acute capacitive-coupling stimulation effectiveness. (**A**) Compound action potential (CAP) induced by capacitive-coupling stimulation of the rat sciatic nerves. (**B**) Compound muscle action potential (CMAP) induced by capacitive-coupling stimulation of the rat sciatic nerves. (**C**) Gastrocnemius muscle tension induced by capacitive coupling of the rat sciatic nerves.

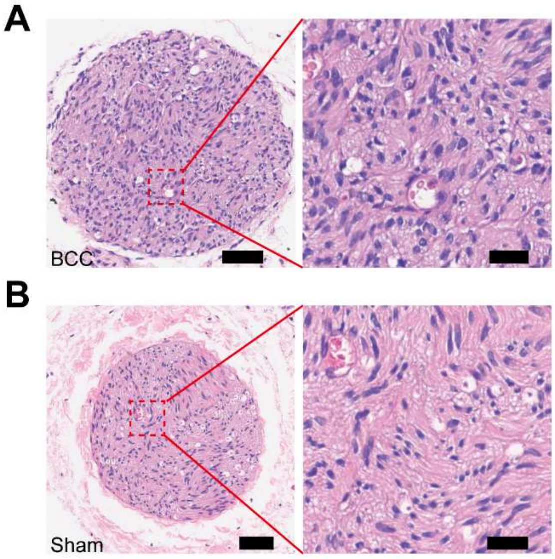

Fig. S16. Histological analysis of vagus nerves at device-implanted sites. (**A**, **B**) H&E staining of the vagus nerve dissected in the BCC (**A**) and sham (**B**) group. The images were magnified ×2.5 (right) to show red regions of the vagus nerve. Scale bars, 50 μm (left), 20 μm (right).

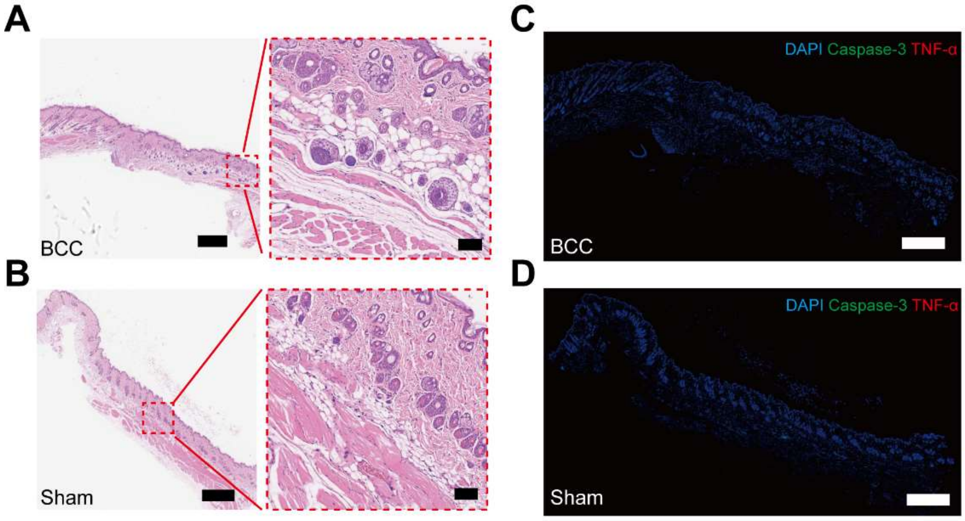

Fig. S17. Histological analysis of skin tissues at device-implanted sites. (**A**, **B**) H&E staining of skin tissues from the BCC and sham groups with a ×10 magnified view (right) highlighting red regions in the skin tissues. Scale bars, 1 mm (left), 100 μm (right). (**C**, **D**) Representative immunofluorescence staining of the skin tissues after 4-week implantation. Scale bar, 1 mm.

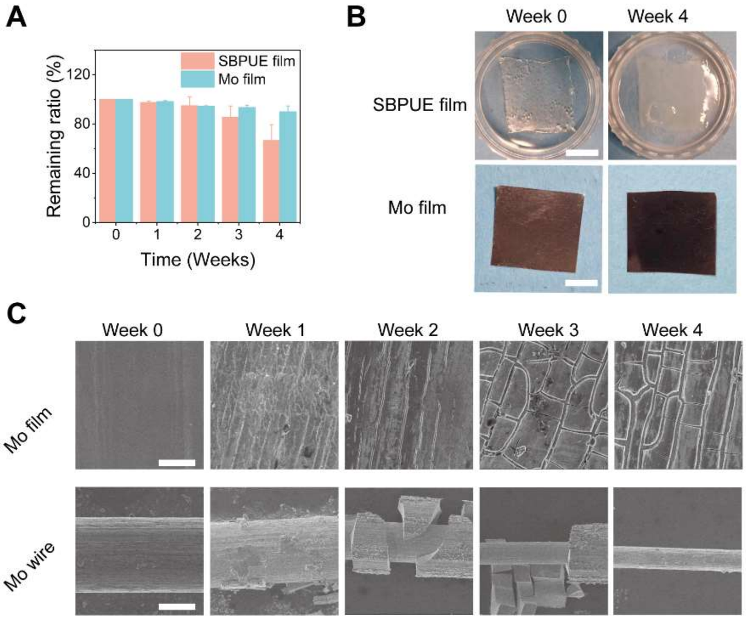

Fig. S18. Biodegradability of the BCC neurostimulator. (**A**) Weight changes of the Mo and SBPUE films after 4-week immersion in 1× PBS at 37 °C (n = 5). (**B**) Images of the Mo and SBPUE films before and after 1-month in vitro degradation. Scale bar, 5 mm. (**C**) SEM images of Mo films and Mo wires after degradation. Scale bar, 50 µm.

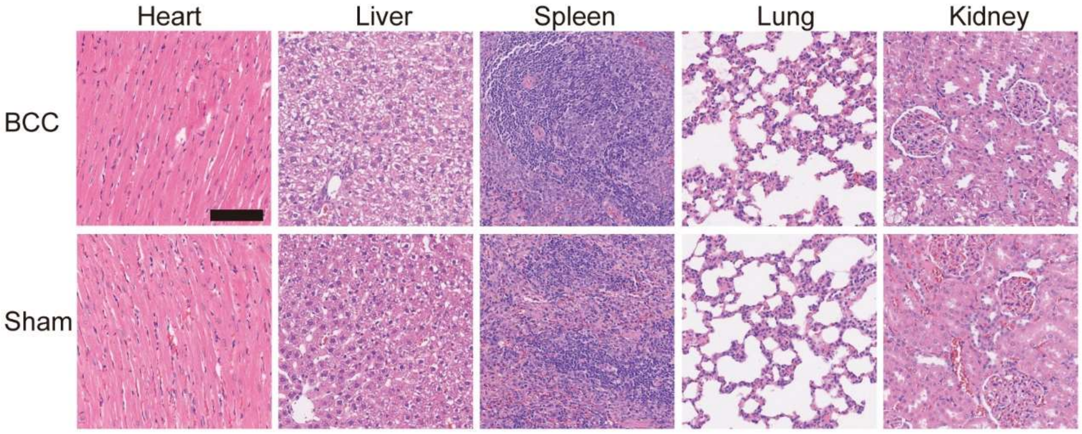

Fig. S19. Histological analysis of major organs (n = 6). H&E staining of the heart, liver, spleen, lung, and kidney tissues in the VNS and Sham group. Scale bar, 100 μm.

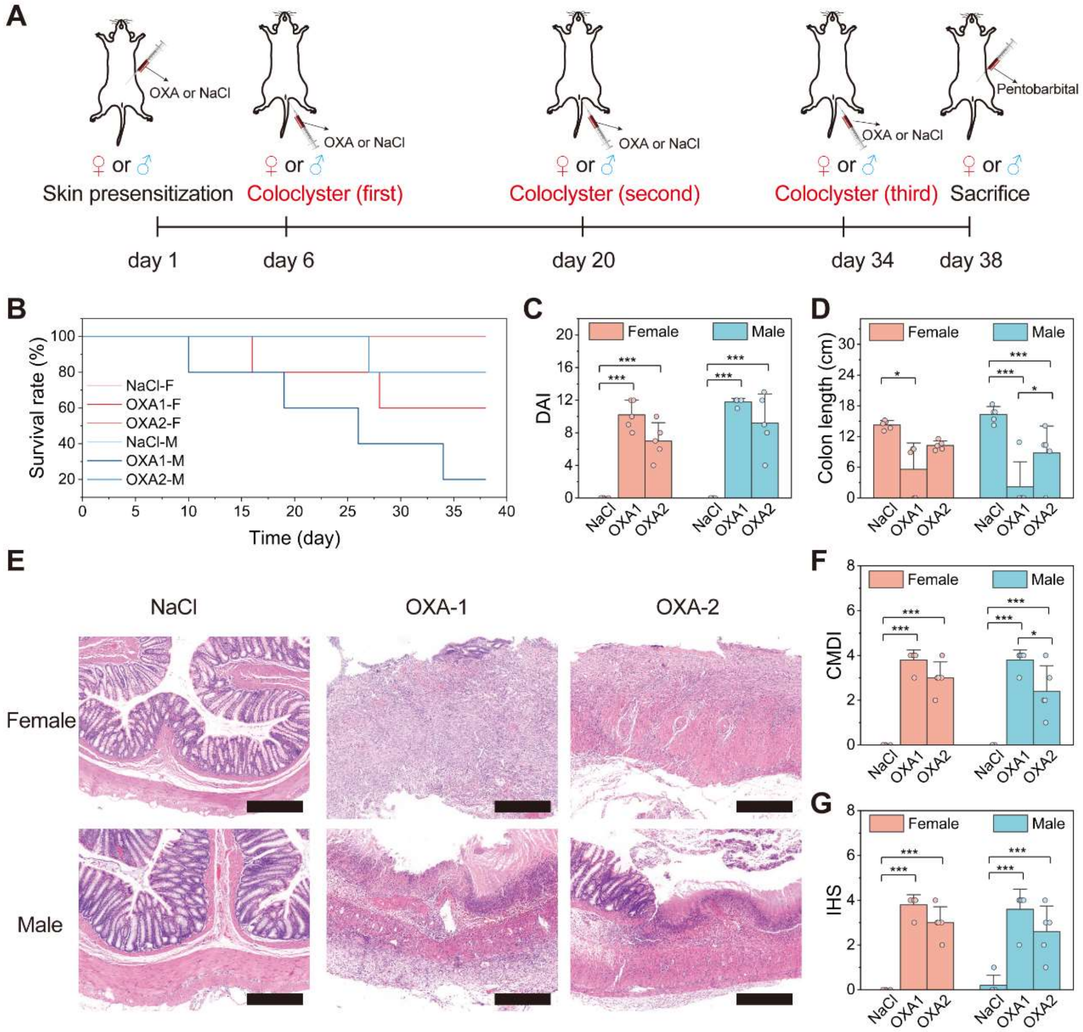

Fig. S20 Effects of gender and enema OXA dose on PIBD rats. (A) Schematic diagram of the OXA-induced PIBD modeling process. **(B)** Survival curves of rats in the different groups (n=5). **(C)** Disease activity index (DAI) score in the different groups (n=5). **(D)** Colon length in the different groups (n=5). **(E)** Representative H&E staining images of rats' colons in the different groups. Scale bar, 500 μm. **(F)** Colon Mucosal Damage Index (CMDI) of rats' colons in the different groups (n=5). **(G)** Inflammation-related histology score (IHS) of rats′ colons in the different groups (n=5). Data are presented as the mean ± standard deviation in (C, D, F, G) and were analyzed by one-way ANOVA first, and then by the Tukey′s post hoc test. *P ≤ 0.05, ***P ≤ 0.001.

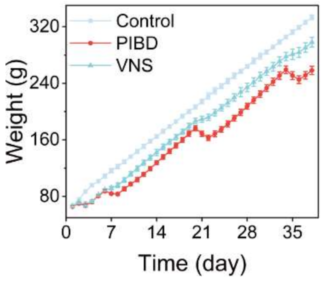

Fig. S21. Weight changes of rats in different groups (n = 5). Data are presented as the mean ± standard deviation.

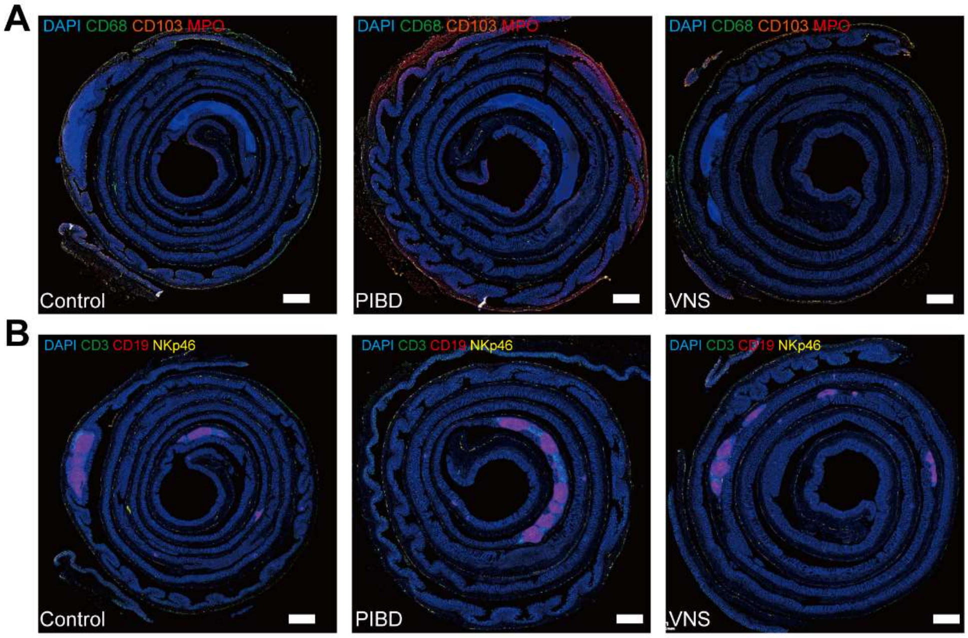

Fig. S22. Representative immunofluorescence staining of the colons from different groups. (**A**) Representative immunofluorescence photographs of adaptive immune cells in the colons from different groups. CD19 and NKp46 were chosen to label B and NK cells, respectively. Scale bar, 1 mm. (**B**) Representative immunofluorescence photographs of innate immune cells in the colons from different groups. CD68, CD103, MPO were chosen to label macrophage, DC, and granulocyte cells, respectively. Scale bar, 1 mm.

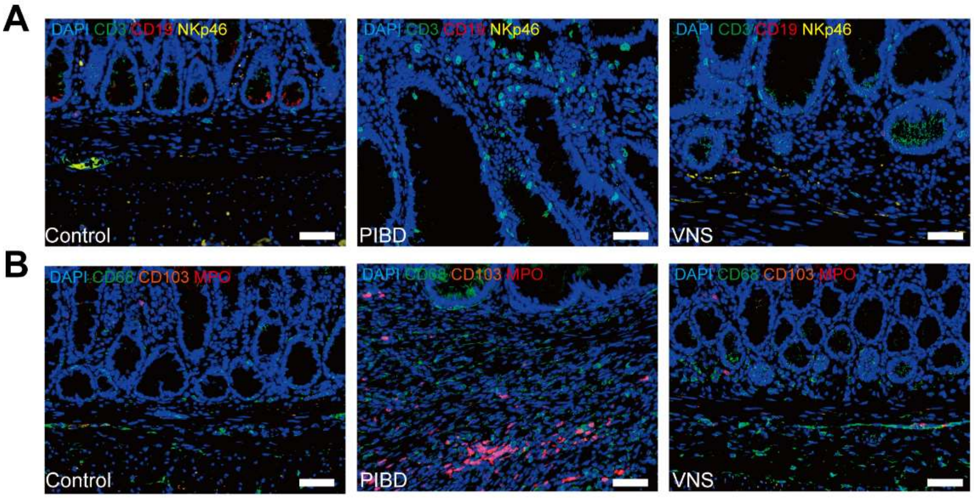

Fig. S23. Immunofluorescence staining of the colons from different groups. (**A**) Representative immunofluorescence photographs of adaptive immune cells in the colons from different groups. Scale bar, 50 µm. (**B**) Representative immunofluorescence photographs of innate immune cells in the colons from different groups. Scale bar, 50 µm. (Fig. 5A shows representative immunofluorescence staining of T cells in the colon from different groups, specifically emphasizing $CD3^+$ T cells. In contrast, fig. S23 includes staining with additional channels, presenting a merged image of CD3, CD19, and NKp46.)

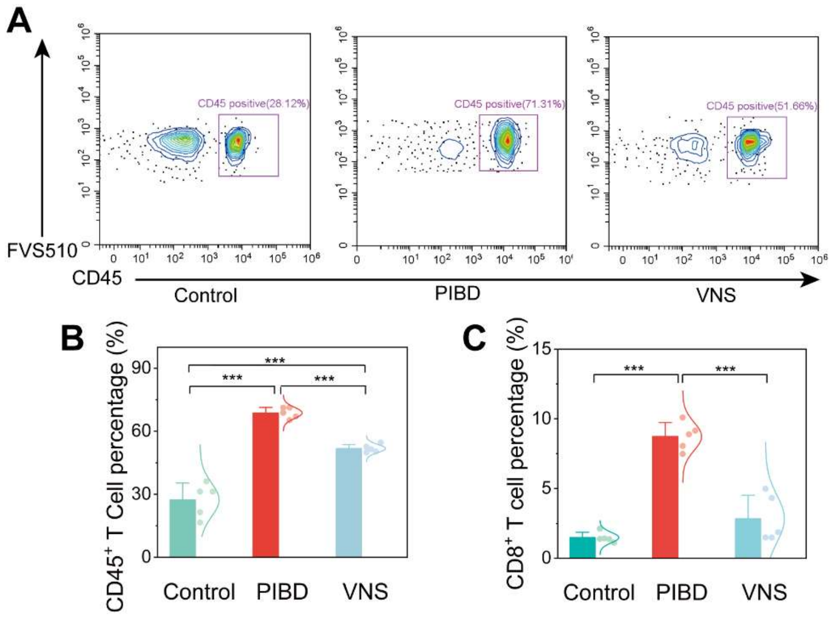

Fig. S24. Flow cytometry and statistical analysis of CD45$^+$ T cells in colons. (**A**) Flow cytometry analysis of CD45$^+$ T cells in the different groups. (**B**) Statistical analysis of CD45$^+$ T cells in the different groups (n = 5). (**C**) Statistical analysis of CD8$^+$ T cells in the different groups (n = 5). Data are presented as the mean ± standard deviation in (**B** and **C**) and were analyzed by one-way ANOVA first, and then by the Tukey′s post hoc test. ***P ⩽ 0.001.

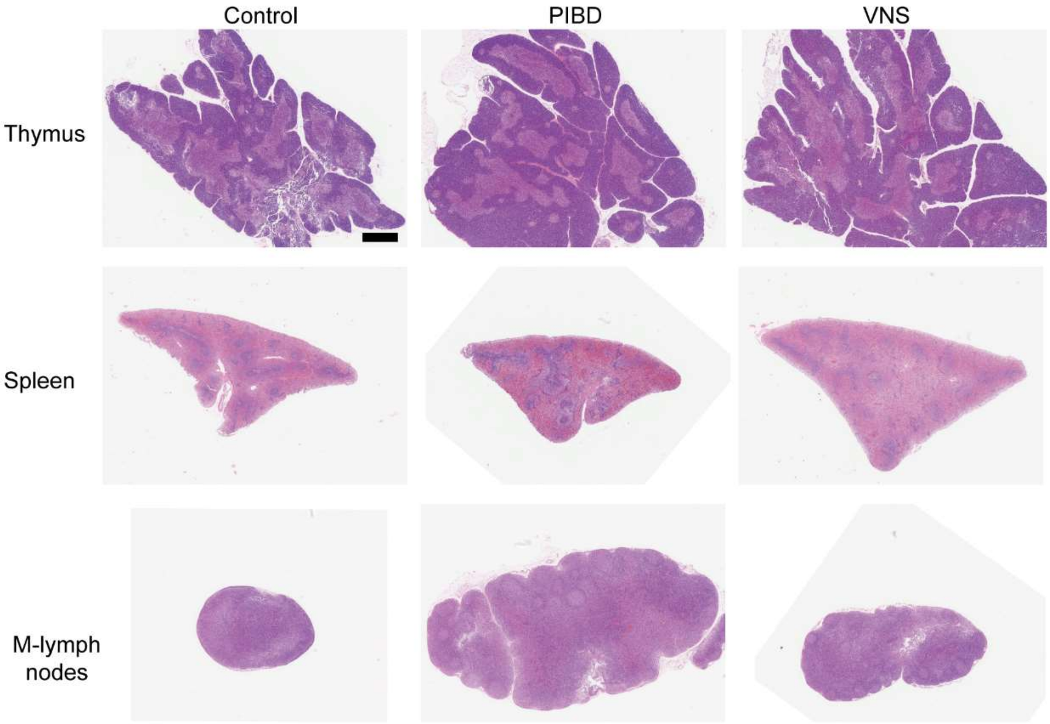

Fig. S25. HE staining of immune organs from different groups. PIBD caused thymic cortical thickening, spleen white pulp expansion, and mesenteric lymph node enlargement, and electroceuticals treatment partially alleviated these symptoms. Scale, 1 mm.

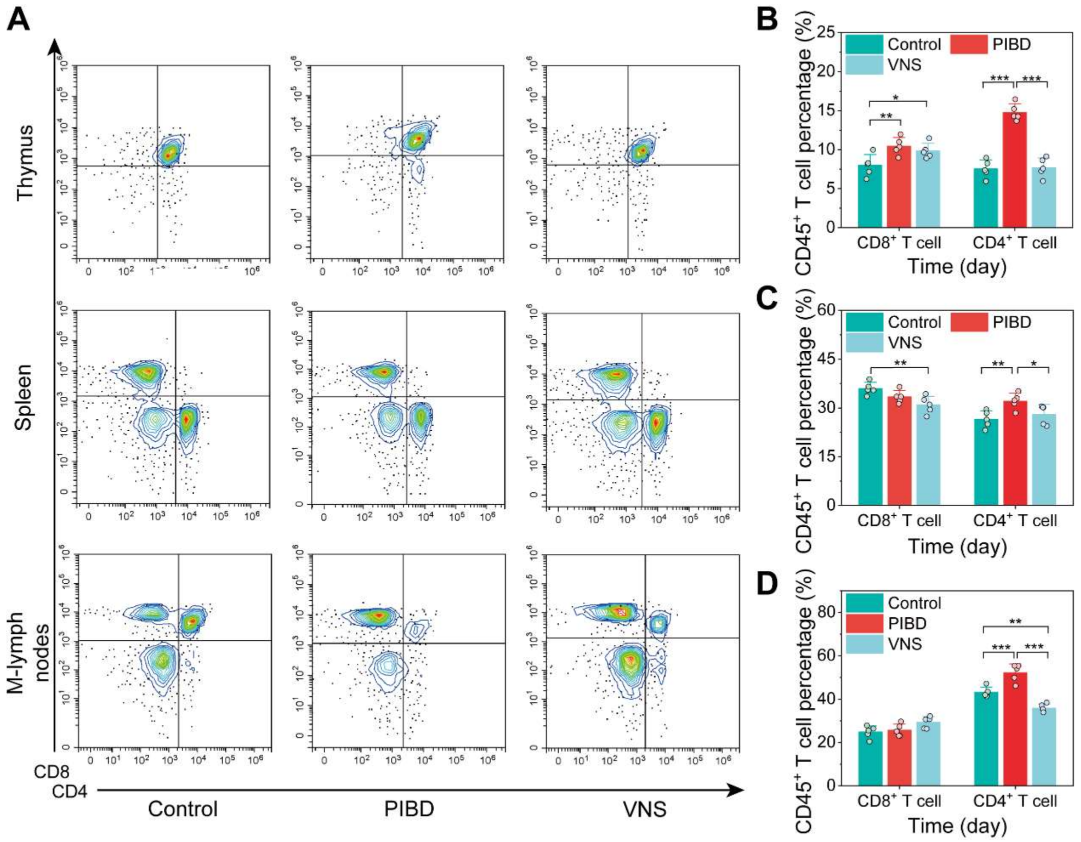

Fig. S26. Flow cytometry and statistical analysis of immune organs. (**A**) Flow cytometry analysis of immune organs in different groups. (**B**-**D**) Statistical analysis of $CD4^+$ and $CD8^+$ T cells in the thymus (**B**), in the spleen (**C**), and in the mesenteric lymph nodes (**D**). Data are presented as the mean ± standard deviation in (**B**, **C**, and **D**) and were analyzed by one-way ANOVA first, and then by the Tukey′s post hoc test. *P ⩽ 0.05, ***P ⩽ 0.001. NS, not significant.

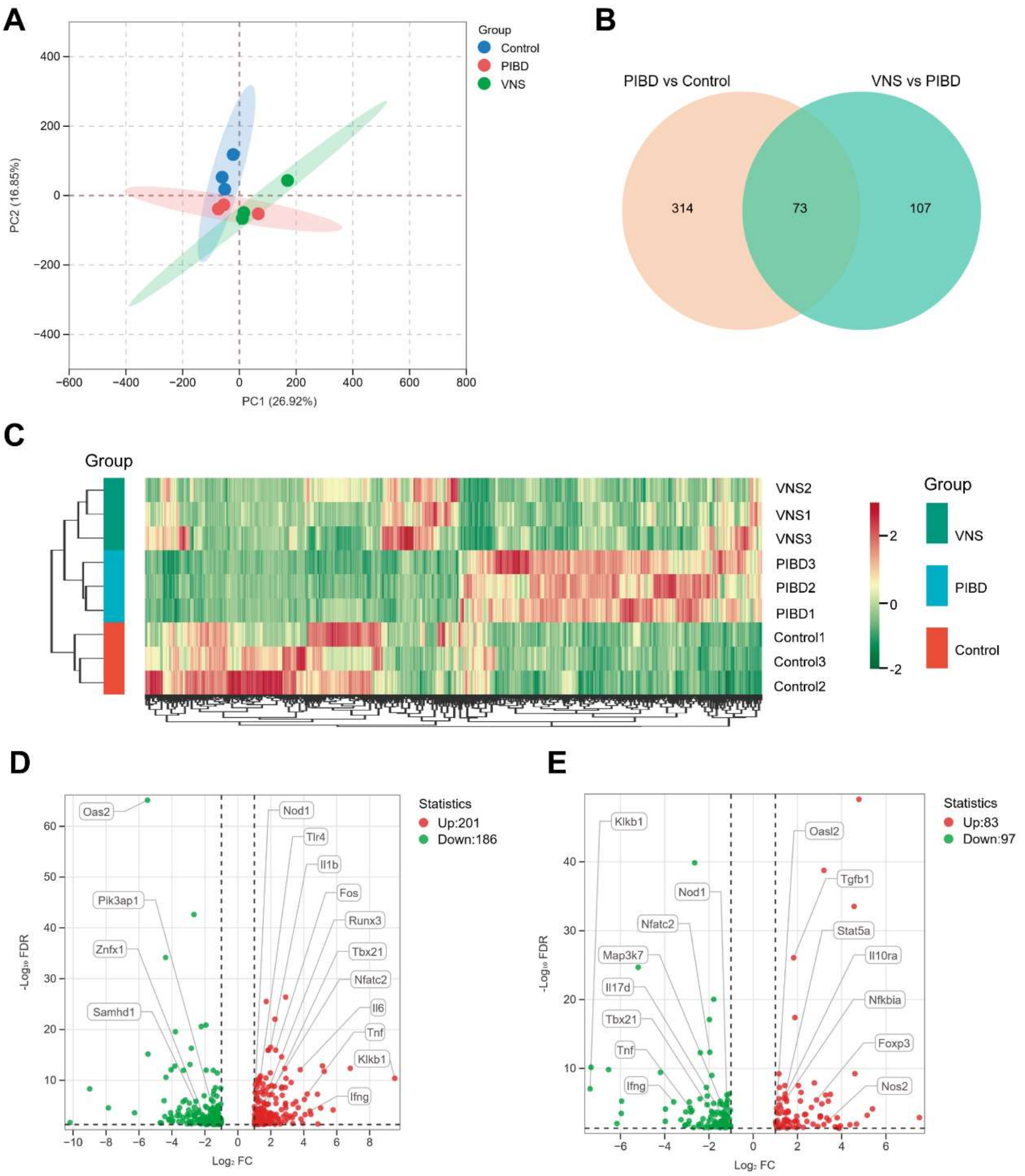

Fig. S27. Bioinformatics analysis of RNA sequencing of rat colons. (**A**) Principal component analysis (PCA) diagram. (**B**) Venn diagram of differentially expressed genes. (**C**) Cluster heat map of differentially expressed genes. (**D**) Volcano plot of differential genes between the PIBD and the control group. (**E**) Volcano plot of differential genes between the VNS and the PIBD group.

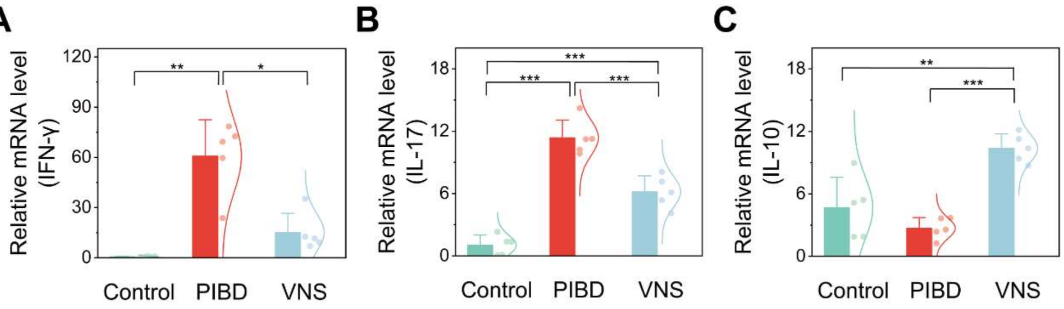

Fig. S28. mRNA levels of major pro-inflammatory and anti-inflammatory cytokines in the guts. (**A**) Relative mRNA of IFN-γ cytokine in the colons (n = 5). (**B**) Relative mRNA of IL-17 cytokine in the colons (n = 5). (**C**) Relative mRNA of IL-4 cytokine in the colons (n = 5). Data are presented as the mean ± standard deviation and were analyzed by one-way ANOVA first, and then by the Tukey′s post hoc test. *P ⩽ 0.05, **P ⩽ 0.01, ***P ⩽ 0.001. NS, not significant.

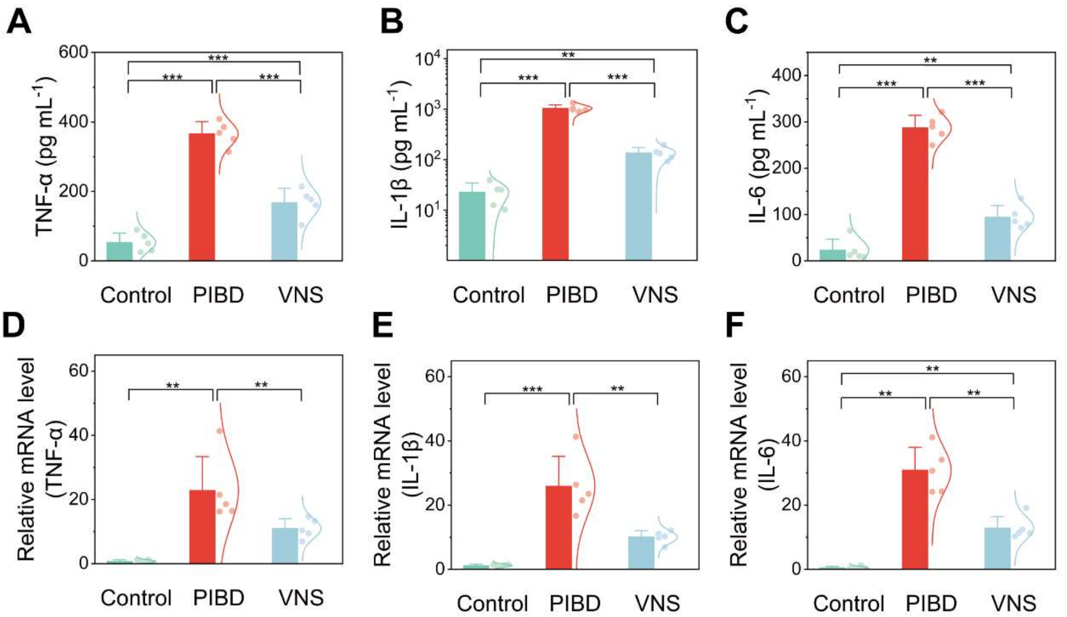

Fig. S29. Concentration and mRNA levels of pro-inflammatory cytokines in the guts. (**A**) Concentration of TNF-α cytokine in the colons (n = 5). (**B**) Concentration of IL-1β cytokine in the colons (n = 5). (**C**) Concentration of IL-6 cytokine in the colons (n = 5). (**D**) Relative mRNA of TNF-α cytokine in the colons (n = 5). (**E**) Relative mRNA of IL-1β cytokine in the colons (n = 5). (**F**) Relative mRNA of IL-6 cytokine in the colons (n = 5). Data are presented as the mean ± standard deviation and were analyzed by one-way ANOVA first, and then by the Tukey′s post hoc test. **P ⩽ 0.01, ***P ⩽ 0.001. NS, not significant.

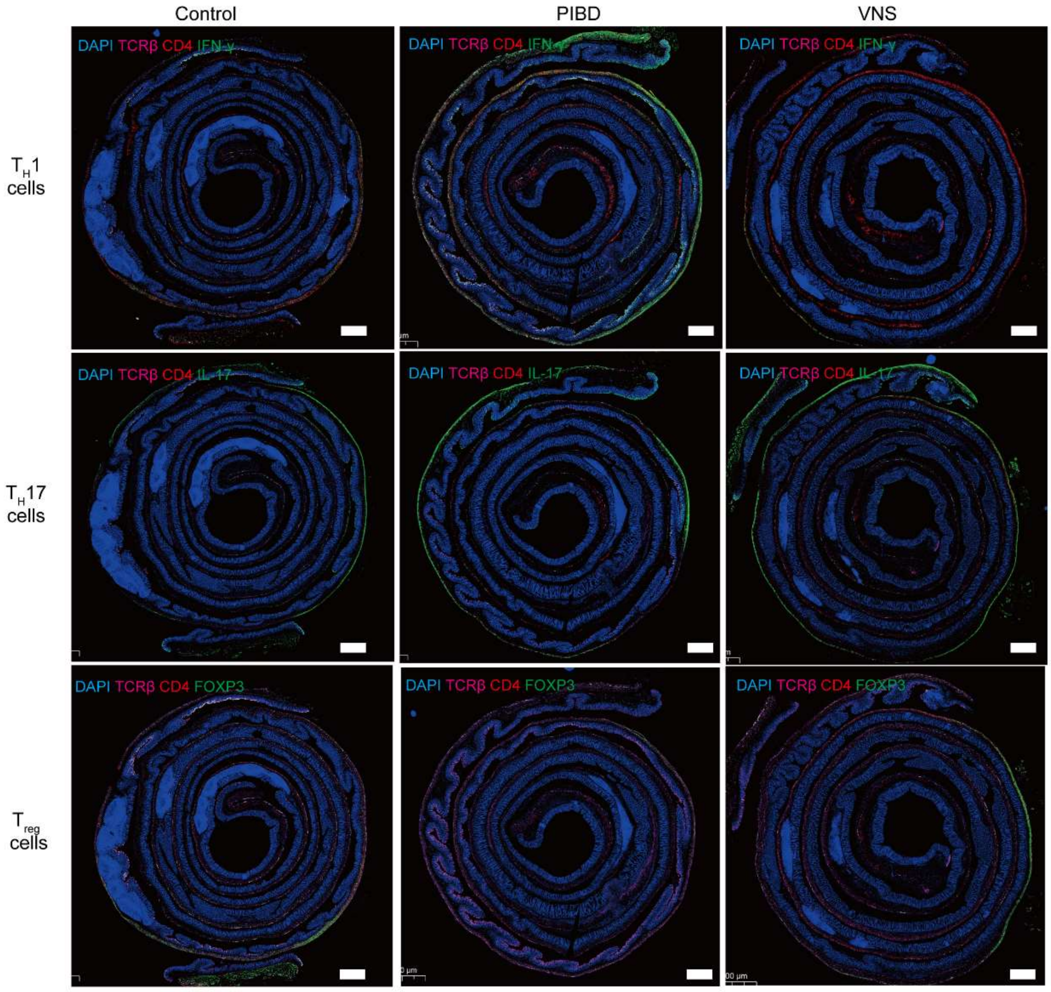

Fig. S30. Immunofluorescence staining of the guts from different groups. TCRβ and CD4 were used to label $CD4^+$ T cells. IFN-γ was used as a marker for T_H1 cells. IL-17 was selected to label T_H17 cells, associated with autoimmune and inflammatory processes. FOXP3 was used to identify Treg cells, which are critical for maintaining immune tolerance and suppressing inflammation. Scale bar, 1 mm. Scale bar, 1 mm. (The groups in fig. S30 consist of three consecutive sections from the same paraffin-embedded colon block, each subjected to different immunofluorescence stains.)

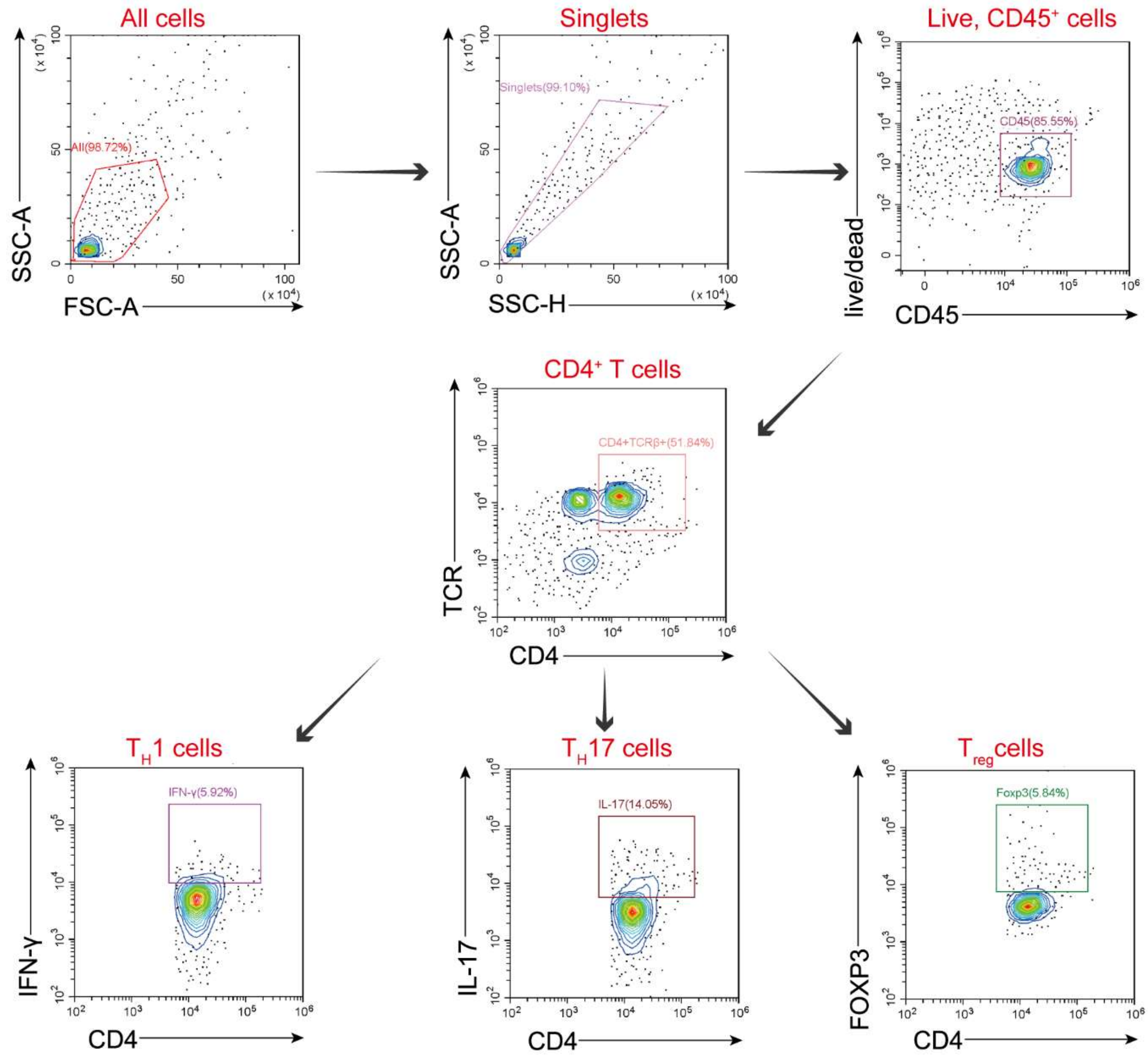

Fig. S31. Flow cytometry analysis process of CD4$^+$ T cell subtypes. The process involved sorting of the CD45$^+$ immune cells, sorting of the CD4$^+$TCRβ$^+$ T cells, and further isolated the IFN-γ$^+$ T$_H$1, IL-17$^+$ T$_H$17, and FOXP3$^+$ T$_{reg}$ cells.

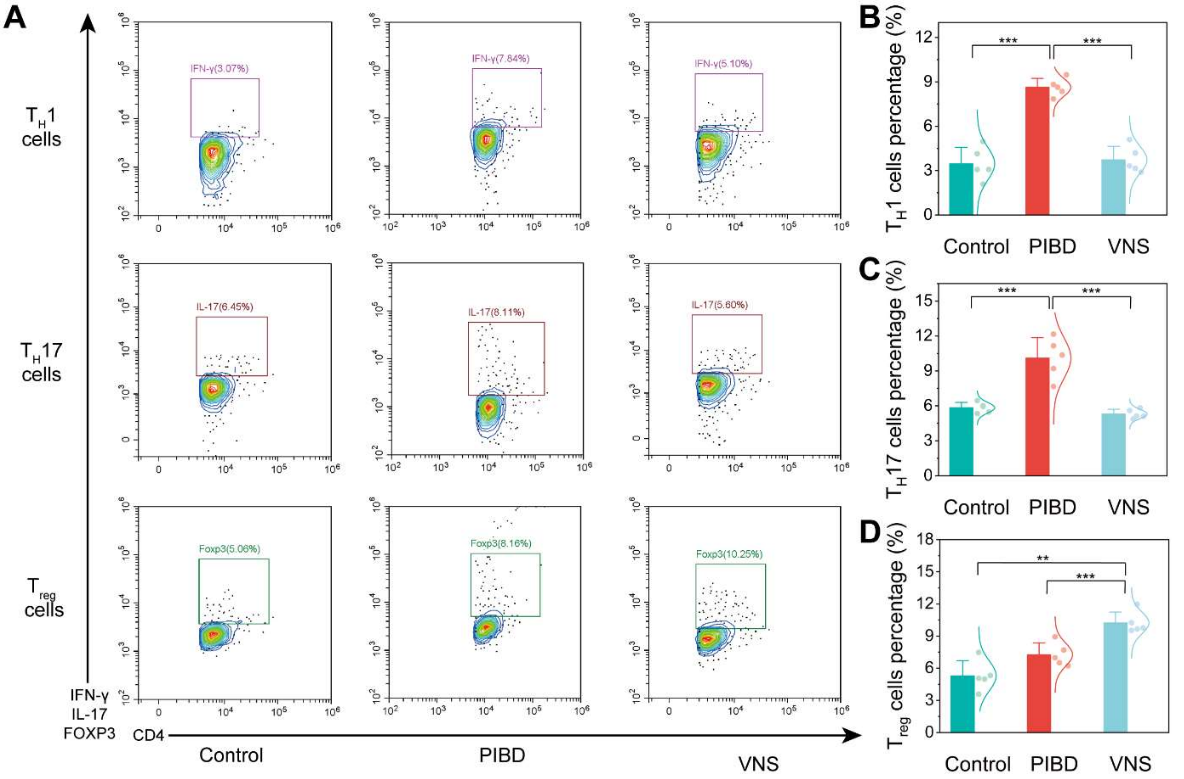

Fig. S32. Flow cytometry of CD4$^+$ T cell subtypes (thymus). FACS plots (**A**) and statistical analysis (**B**, **C**, **D**) of CD4$^+$ T cell subtypes (n = 5). Data are presented as the mean ± standard deviation in (**B**, **C**, and **D**) and were analyzed by one-way ANOVA first, and then by the Tukey′s post hoc test. **P ≤ 0.01, ***P ≤ 0.001. NS, not significant.

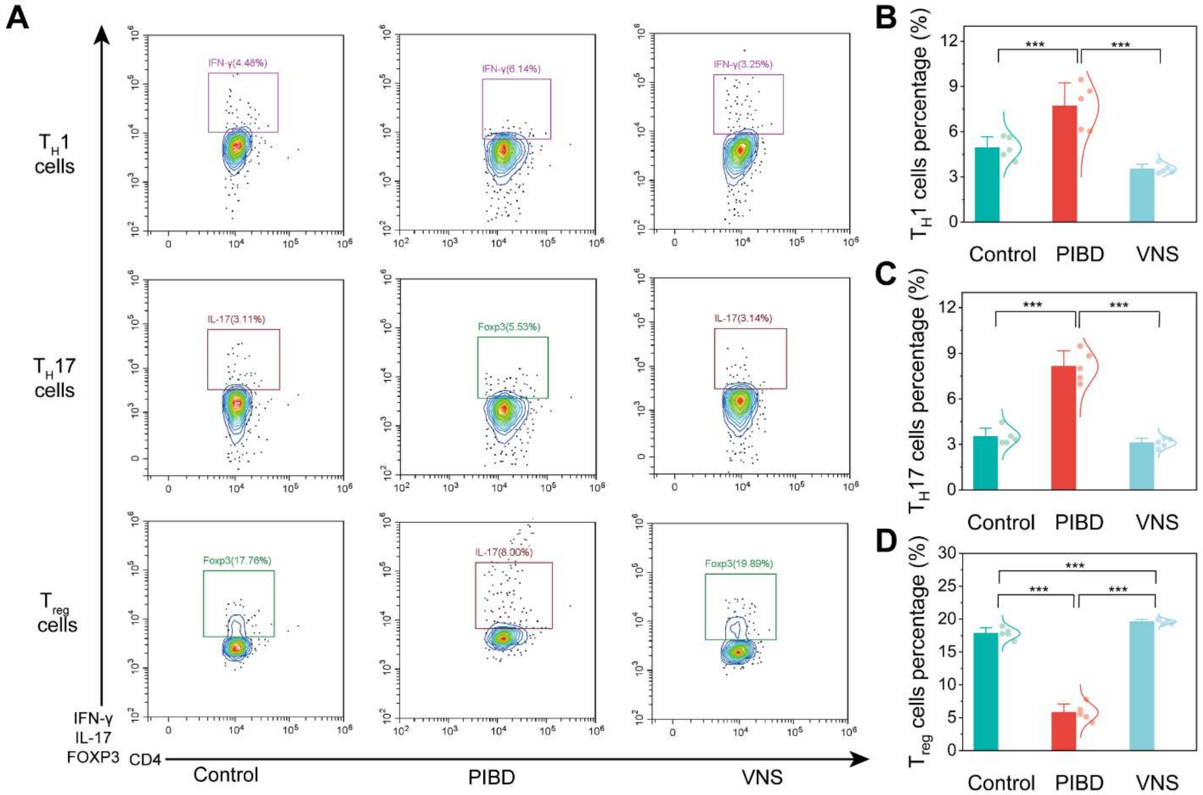

Fig. S33. Flow cytometry of CD4⁺ T cell subtypes (spleen). FACS plots (**A**) and statistical analysis (**B**, **C**, **D**) of $CD4^+$ T cells subtypes (n = 5). Data are presented as the mean ± standard deviation in (**B**, **C**, and **D**) and were analyzed by one-way ANOVA first, and then by the Tukey′s post hoc test. ***P ≤ 0.001. NS, not significant.

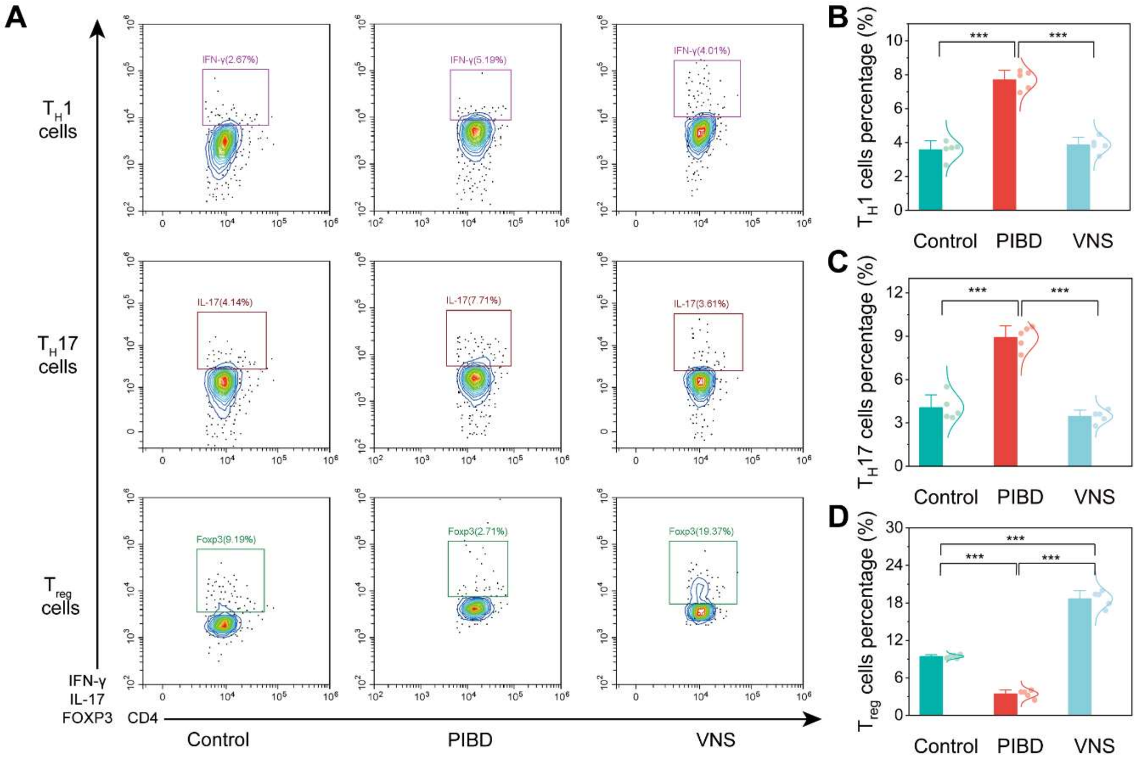

Fig. S34. Flow cytometry of CD4$^+$ T cell subtypes (Lymph nodes). FACS plots (**A**) and statistical analysis (**B**, **C**, **D**) of CD4$^+$ T cell subtypes (n = 5). Data are presented as the mean ± standard deviation in (**B**, **C**, and **D**) and were analyzed by one-way ANOVA first, and then by the Tukey′s post hoc test. ***P ≤ 0.001. NS, not significant.

Table S1. The primer sequences for RT-qPCR.

Primer	Species	Primer sequences 5′-3′
Actin (forward)	Rattus	CTGTGCTATGTTGCCCTAGACTTC
Actin (reverse)	Rattus	GAACCGCTCATTGCCGATAGTG
TNF-α (forward)	Rattus	CACGCTCTTCTGTCTACTGAACTTC
TNF-α (reverse)	Rattus	TTGCCCTCCCACCCTACTTTG
IFN-γ (forward)	Rattus	GACTAATCAAAGAGGAAGGTGGTAGAC
IFN-γ (reverse)	Rattus	TGAACAGATGGTAACTCCGAACTTG
IL-1β (forward)	Rattus	GTTCATCATCAATCCTCAGTCCTCTC
IL-1β (reverse)	Rattus	TTGTTGTTGTTGTTGTCACATCTCC
IL-4 (reverse)	Rattus	TGTTGCTCCGCTCCACTTACC
IL-6 (forward)	Rattus	CTTCCAGCCAGTTGCCTTCTTG
IL-6 (reverse)	Rattus	TGGTCTGTTGTGGGTGGTATCC
IL-10 (forward)	Rattus	ACCAGAAGGAGCAGGAAGCAG
IL-10 (reverse)	Rattus	GAATAGGTTCACAGGCAGAAGGAAG
IL-17A (forward)	Rattus	TTGCTGATTGAGACCAGGTTGTG
IL-17A (reverse)	Rattus	GGCATCTATCAAAGGTTTCATTTCCAG

Movie S1. Capacitive-coupling electrical-stimulation pulses induce sciatic nerve response
